# Comparative genomics incorporating translocation renal cell carcinoma mouse model reveals molecular mechanisms of tumorigenesis

**DOI:** 10.1172/JCI170559

**Published:** 2024-02-22

**Authors:** Gopinath Prakasam, Akhilesh Mishra, Alana Christie, Jeffrey Miyata, Deyssy Carrillo, Vanina T. Tcheuyap, Hui Ye, Quyen N. Do, Yunguan Wang, Oscar Reig Torras, Ramesh Butti, Hua Zhong, Jeffrey Gagan, Kevin B. Jones, Thomas J. Carroll, Zora Modrusan, Steffen Durinck, Mai-Carmen Requena-Komuro, Noelle S. Williams, Ivan Pedrosa, Tao Wang, Dinesh Rakheja, Payal Kapur, James Brugarolas

**Affiliations:** 1 Kidney Cancer Program, Simmons Comprehensive Cancer Center,; 2 Hematology-Oncology Division, Department of Internal Medicine,; 3 Peter O’ Donnell Jr. School of Public Health,; 4 Department of Radiology, and; 5 Quantitative Biomedical Research Center, Department of Population and Data Sciences, The University of Texas Southwestern Medical Center, Dallas, Texas, USA.; 6 Department of Medical Oncology and Translational Genomics and Targeted Therapies in Solid Tumors, Institut d’Investigacions Biomèdiques August Pi I Sunyer (IDIBAPS), Hospital Clinic de Barcelona, Barcelona, Spain.; 7 Department of Pathology, The University of Texas Southwestern Medical Center, Dallas, Texas, USA.; 8 Department of Orthopaedics and Oncological Sciences, Huntsman Cancer Institute, University of Utah, Salt Lake City, Utah, USA.; 9 Department of Molecular Biology and Department of Internal Medicine, The University of Texas Southwestern Medical Center, Dallas, Texas, USA.; 10 Department of Microchemistry, Proteomics, Lipidomics and Next Generation Sequencing and; 11 Department of Oncology Bioinformatics, Genentech Inc., South San Francisco, California, USA.; 12 Department of Biochemistry,; 13 Advanced Imaging Research Center, and; 14 Department of Urology, The University of Texas Southwestern Medical Center, Dallas, Texas, USA.

**Keywords:** Cell biology, Oncology, Cancer, Molecular genetics, Mouse models

## Abstract

Translocation renal cell carcinoma (tRCC) most commonly involves an *ASPSCR1-TFE3* fusion, but molecular mechanisms remain elusive and animal models are lacking. Here, we show that human *ASPSCR1-TFE3* driven by Pax8-Cre (a credentialed clear cell RCC driver) disrupted nephrogenesis and glomerular development, causing neonatal death, while the clear cell RCC failed driver, Sglt2-Cre, induced aggressive tRCC (as well as alveolar soft part sarcoma) with complete penetrance and short latency. However, in both contexts, ASPSCR1-TFE3 led to characteristic morphological cellular changes, loss of epithelial markers, and an epithelial-mesenchymal transition. Electron microscopy of tRCC tumors showed lysosome expansion, and functional studies revealed simultaneous activation of autophagy and mTORC1 pathways. Comparative genomic analyses encompassing an institutional human tRCC cohort (including a hitherto unreported *SFPQ-TFEB* fusion) and a variety of tumorgraft models (*ASPSCR1-TFE3*, *PRCC-TFE3*, *SFPQ-TFE3*, *RBM10-TFE3*, and *MALAT1-TFEB*) disclosed significant convergence in canonical pathways (cell cycle, lysosome, and mTORC1) and less established pathways such as Myc, E2F, and inflammation (IL-6/JAK/STAT3, interferon-γ, TLR signaling, systemic lupus, etc.). Therapeutic trials (adjusted for human drug exposures) showed antitumor activity of cabozantinib. Overall, this study provides insight into MiT/TFE-driven tumorigenesis, including the cell of origin, and characterizes diverse mouse models available for research.

## Abstract 2

Characterization of a human data and mouse models reveals *molecular mechanisms of ASPSCR1-TFE3-mediated transformation* in renal cell carcinoma.

## Graphical abstract

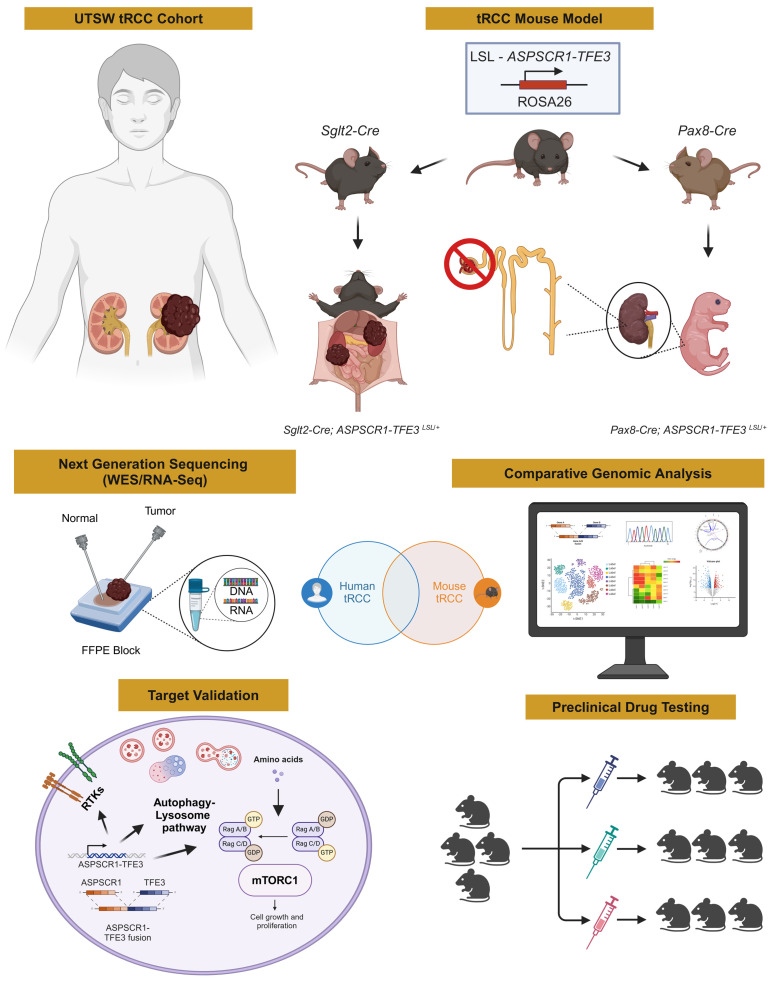

## Introduction

Renal cell carcinoma (RCC) is the most common malignant kidney epithelial neoplasm. Translocation renal cell carcinoma (tRCC) is an aggressive molecular subtype unusually prevalent in children and adolescents (1, 2). It is estimated that tRCC accounts for about 5% of all RCC, but the prevalence is likely underestimated owing to overlapping histological features and the need for molecular testing to confirm the diagnosis (2, 3). tRCC has a poor prognosis with no specific therapeutic options and is incurable in the metastatic setting.

tRCC is characterized by chromosomal translocations involving 3 genes of the MiT family of transcription factors: *TFE3* (Xp11.23), *TFEB* (6p21.1), and *MITF* (3p13) (4–7). The most commonly translocated gene is *TFE3*, and over a dozen partners have been identified (8), including *ASPSCR1* t(X;17) (p11.23; q25.3) (9), *SFPQ* t(X;1) (p11.23; p34.3) (10), and *PRCC* t(X;1) (p11.23; q23.1) (5). The most common gene fusion is *ASPSCR1-TFE3*. Notably, *ASPSCR1-TFE3* is also implicated in the pathogenesis of alveolar soft part sarcoma (ASPS), a rare soft tissue sarcoma (11, 12), which, like tRCC, may present in children and young adults (13). ASPSCR1-TFE3, along with other TFE3 fusion proteins, also contributes to the development of a subset of perivascular epithelioid cell tumors (PEComas) (14, 15). Interestingly, these PEComas are characterized by the absence of more common mutations in the *TSC1* and *TSC2* genes, which also alter TFE3/TFEB regulation, upon which the pathogenesis may converge (16–18).

MiT/TFE family members are characterized by a basic helix-loop-helix (bHLH) DNA-binding domain and a leucine zipper dimerization domain. They recognize a subtype of E-box, referred to as the CLEAR sequence. MiT/TFE family members functionally overlap but also exhibit independent functions by regulating particular gene sets (19). Collectively, they regulate catabolism, specifically by promoting autophagy and lysosomal biogenesis (12). Our current understanding of their role in tumorigenesis points to aberrant constitutive expression and nuclear localization of transcriptionally active chimeric fusion proteins that preserve the MiT DNA-binding and dimerization domains (6). However, how these fusion proteins induce tRCC is poorly understood.

Advances in our understanding of tRCC biology have been hampered by a paucity of animal models. One patient-derived xenograft (PDX) model with an *SFPQ-TFE3* translocation has previously been reported (20), and there have been several attempts to develop genetically engineered mouse models (GEMMs). However, to date, tRCC GEMMs are limited by the development of cysts and small tumors that do not recapitulate the aggressiveness of the human disease (21, 22).

Here, we report genomic analyses of an institutional human tRCC cohort (including tRCC PDX, also called tumorgraft [TG], models) and comparative genomic studies with a novel tRCC mouse model we generated. We show that conditional expression of a human ASPSCR1-TFE3 fusion protein using a *Pax8-Cre* driver, which we previously showed drives a variety of clear cell RCC (ccRCC) oncogenotypes (23), disrupted nephrogenesis and glomerular development, causing neonatal death. In contrast, ASPSCR1-TFE3 expression in the *Sglt2-Cre* lineage led to tRCC with complete penetrance and short latency. Histopathological studies, including immunohistochemistry (IHC) and transmission electron microscopy, showed that murine tRCC recapitulates the features of the human disease. Interestingly, *Sglt2-Cre;* *ASPSCR1-TFE3* mice also developed ASPS. Comparative genomic analyses disclosed significant convergence in canonical pathways (cell cycle, lysosome, and mTORC1) and less established pathways such as Myc, E2F, p53, and inflammation (interferon-γ [IFN-γ] response, IL-6/JAK/STAT3, TLR signaling, systemic lupus, and NK cytotoxicity). Integration of transcriptomic analyses with previously published ChIP-Seq data uncovered several putative novel direct ASPSCR1-TFE3 targets. Along with mTORC1 activation, we observed aberrant overexpression of the receptor tyrosine kinases *MET* and *RET*. Drug trials with cabozantinib, which inhibits both MET and RET, disrupted tRCC tumor growth in mice. Overall, our study provides biological insights into MiT/TFE gene fusion–driven transcriptional programs that dictate tRCC tumorigenesis and significantly expands the available tumor models (both GEMMs and TGs) for research and therapeutic development.

## Results

### Genomic characterization of human tRCC cohort.

We evaluated patients at The University of Texas Southwestern Medical Center (UTSW) affiliated hospitals, including Parkland Hospital and Children’s Medical Center, and report on 30 cases of tRCC including 16 cases not previously published and 4 previously published for which genomics are reported for the first time (7, 24, 25) (Supplemental Tables 1 and 2; supplemental material available online with this article; https://doi.org/10.1172/JCI170559DS1). Clinical diagnosis was based on histology and IHC (pan-cytokeratin, cathepsin K, and Melan A) and was typically confirmed by fluorescence in situ hybridization (FISH) or conventional cytogenetics (Supplemental Table 1). Most *TFE3* translocation cases presented in young individuals (median age, 34). In contrast, *TFEB* gene rearrangement/amplification cases were more common in older patients (median age, 68). Among the 30 cases, 3 patients presented with metastatic disease at initial diagnosis, and subsequent metastases developed in 9 additional patients (Supplemental Table 1).

**Figure 1:**
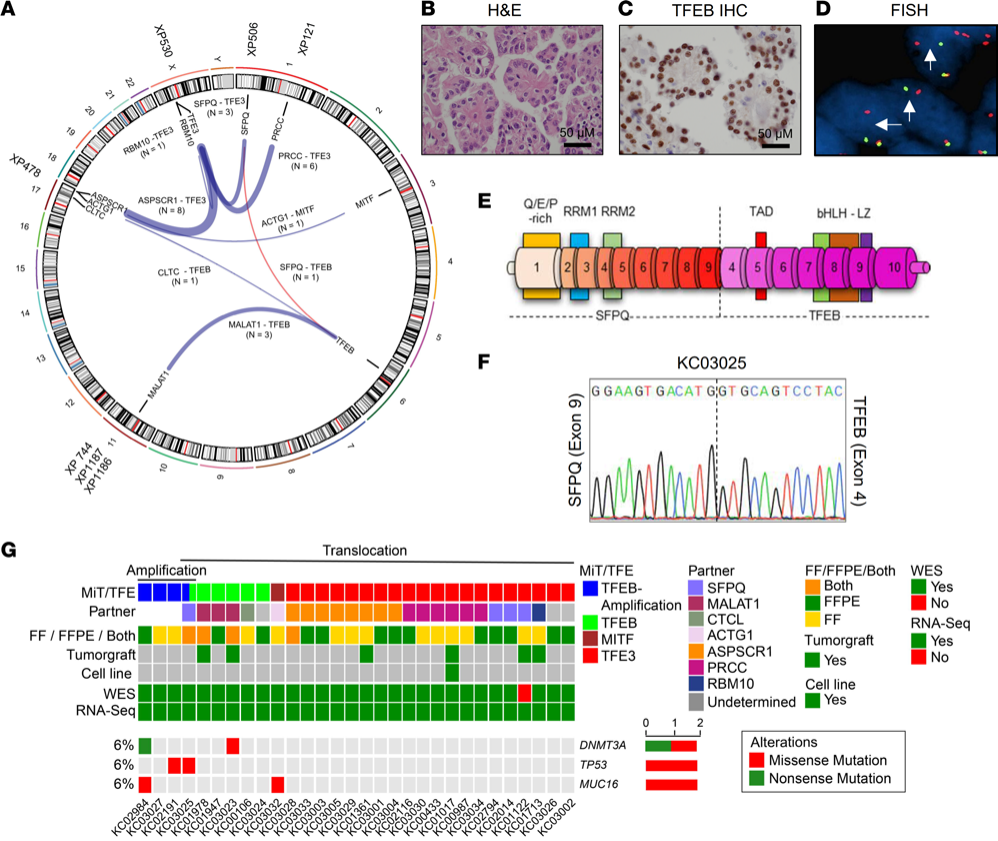
MiT/TFE gene rearrangements and mutational landscape of UTSW tRCC cohort. (**A**) Circos plot with weighted lines for MiT/TFE gene fusions identified by RNA-Seq (*n* = 24). A previously unreported *SFPQ-TFEB* gene fusion t(6;1) (p21.1; p34.3) is shown in red. Where available, tumorgrafts are included in the periphery. (**B**–**D**) Characterization of novel *SFPQ-TFEB* tRCC case (KC03025) by H&E (**B**), TFEB IHC (**C**), and FISH using *TFEB* break-apart probes stained with CytoRed and CytoGreen (**D**). Scale bar: 50 μm (**B** and **C**). (**E**) Illustration of *SFPQ-TFEB* chimeric transcript. RRM, RNA recognition motif; TAD, transcription activation domain; bHLH, basic helix-loop-helix domain; LZ, leucine zipper. (**F**) Sanger sequencing electropherogram (one direction shown) of *SFPQ-TFE3* gene fusion cDNA. (**G**) Oncoprint representation of somatic mutations for COSMIC database genes in tRCC cohort (*n* = 30).

Tumor samples were subjected to RNA-Seq and gene fusion analyses (Supplemental Table 2). We used STAR-Fusion software, and for a few cases where a fusion was not identified, we inspected unaligned discordant reads of MiT/TFE genes using Integrative Genomics Viewer (see Supplemental Methods). Chromosomal breakpoints and partner fusion genes were determined for 24 cases. They included fusions of *TFE3* with *ASPSCR1* (*n* = 8), *PRCC* (*n* = 6), *SFPQ* (*n* = 3), and *RBM10* (*n* = 1). In addition, we found *TFEB* fusions with *MALAT1* (*n* = 3) and *CTCL* (*n* = 1). An *MITF-ACTG1* fusion, which we reported previously (7), was also present (*n* = 1) (Figure 1A and Supplemental Table 3). We identified one translocation, *SFPQ-TFEB* t(6;1) (p21.1; p34.3), which was previously unreported in the literature (Figure 1A). The SFPQ-TFEB tumor had characteristic histological features including cells with high-grade nuclei and abundant eosinophilic and clear cytoplasm arranged in papillary architecture (Figure 1B). Strong nuclear TFEB signal was observed by IHC (Figure 1C). FISH with *TFEB* 5′ and 3′ probes showed a split-apart signal in tumor cells (Figure 1D). Chromosomal breakpoints were mapped to downstream of *SFPQ* exon 9 and upstream of *TFEB* exon 4 (Figure 1E and Supplemental Table 3). The fusion was confirmed by reverse transcriptase PCR using primers for exons flanking the chimeric transcript and bidirectional Sanger sequencing (Figure 1F).

Tumors from a subset of patients in the cohort were implanted in NOD/SCID mice (26–28), and for 6 patients, tumors successfully engrafted, leading to stable TG lines (Supplemental Figure 1). TG lines were generated from 2 patients with a *MALAT1-TFEB* gene fusion (KC01978 and KC03023), in one case from the primary tumor (XP744) and in the other from both the primary tumor and a lymph node metastasis (XP1186 and XP1187, respectively). In addition, TG models were generated from tumors with *RBM10-TFE3* (KC01713; XP530), *SFPQ-TFE3* (KC01122; XP506), *ASPSCR1-TFE3* (KC01361; XP478), and *PRCC-TFE3* (KC01017; XP121). From one TG, we also generated a cell line (KC01017; XP121) (Supplemental Figure 1). We validated the predicted MiT/TFE translocations by reverse transcriptase PCR using primers specific to the exons flanking the chimeric transcripts and by Sanger sequencing. Histological characterization revealed substantial resemblance between TGs and corresponding patient tumors, and gene expression analyses showed clustering of TGs with the respective patients (Supplemental Figure 1).

RNA-Seq analyses of gene fusions were complemented with whole exome sequencing (WES) copy number variation analyses, which identified 4 cases with *TFEB* amplification: KC02984, KC03027, KC02191, and KC03025 (where the *SFPQ-TFEB* fusion was also observed) (Supplemental Figure 2). Taken together, we identified MiT/TFE drivers for 27 of the 30 cases. For 3 cases we were not successful in identifying an MiT/TFE abnormality despite diagnostic confirmation with FISH or cytogenetics in the clinical laboratory (Supplemental Table 1).

Mutation calling analysis of 29 tRCC cases (1 sample failed) revealed several mutated genes included in the Catalogue of Somatic Mutations in Cancer (COSMIC) (Supplemental Table 4). However, after stringent mutation calling thresholds, only 3 genes were mutated in more than 1 tumor (Figure 1G). All 3 genes, *TP53*, *DNMT3A*, and *MUC16*, were previously reported to be mutated in tRCC (29, 30). However, in keeping with previous results, no genes were found that were mutated in a substantial number of tumors.

### Pax8-Cre; ASPSCR1-TFE3 model reveals cell fate alterations and nephrogenesis disruption.

To further understand the pathogenesis of tRCC, we sought to generate a mouse model. We bred mice with a conditional human *ASPSCR1-TFE3* cDNA (type 2 fusion) (31) to mice with kidney-lineage Cre recombinases (23). By recombining a *loxP*-stop-*loxP* (*LSL*) cassette upstream of *ASPSCR1-TFE3* in the *Rosa26* locus, *ASPSCR1-TFE3* could thus be expressed in the mouse kidney. We first deployed a *Pax8-Cre* driver, which has broad nephron expression and can lead to oncogenotypically diverse models of ccRCC (23). Pax8 appears early in embryogenesis and is expressed in proximal and distal renal tubules, loops of Henle, collecting ducts, and parietal epithelial cells of the Bowman capsule.

**Figure 2:**
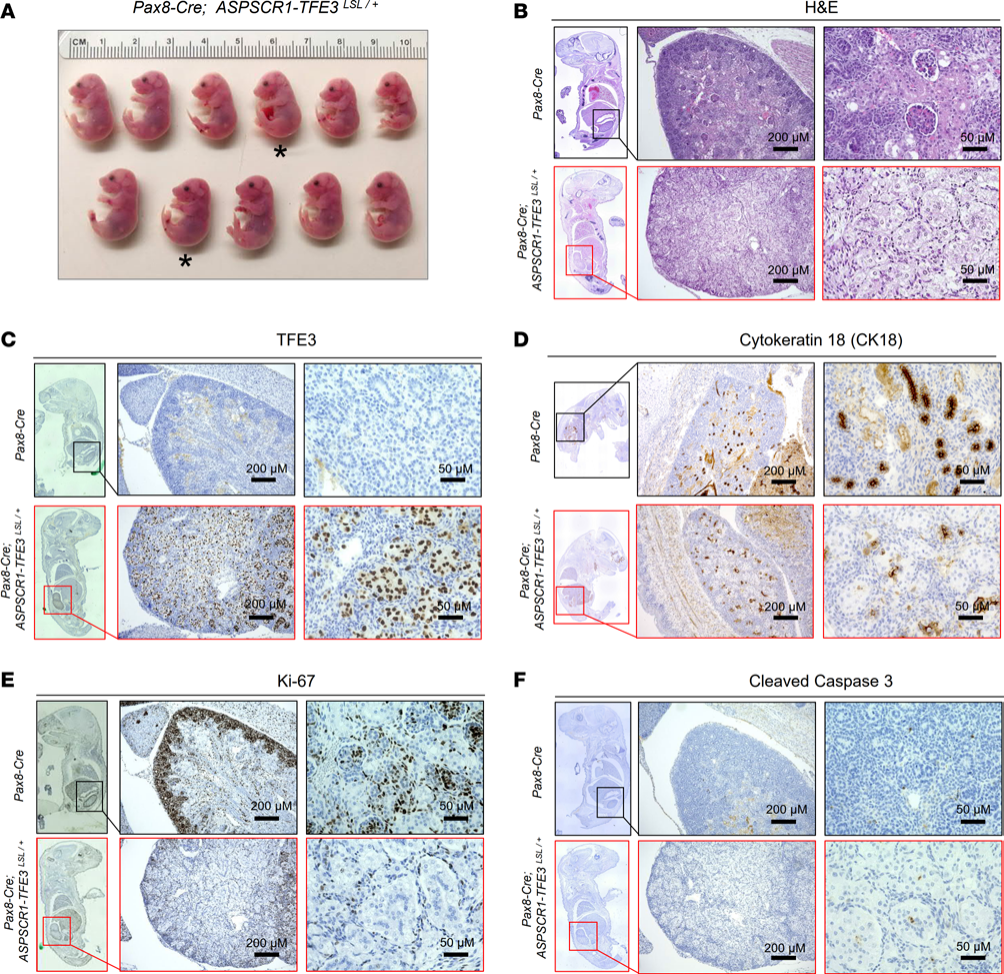
Characterization of the *Pax8-Cre; ASPSCR1-TFE3^LSL/+^* model. (**A**) Macroscopic images of fetuses at E19–20 (*Pax8-Cre; ASPSCR1-TFE3^LSL/+^* fetuses marked by an asterisk). (**B**) H&E-stained images of representative *Pax8-Cre; ASPSCR1-TFE3^LSL/+^* fetuses and littermate controls. (**C**–**F**) IHC for TFE3 (using human-specific antibody), CK18, Ki-67, and cleaved caspase-3 in kidneys from *Pax8-Cre; ASPSCR1-TFE3^LSL/+^* fetuses compared with controls. Scale bars: 200 μm, middle panels; 50 μm, right panels.

Unexpectedly, *Pax8-Cre*–mediated induction of *ASPSCR1-TFE3* resulted in neonatal lethality. No gross anatomic differences between *Pax8-Cre; ASPSCR1-TFE3^LSL/+^* fetuses and littermate counterparts were observed at E19–20 (Figure 2A). However, histological analyses of the kidneys showed that glomeruli were absent in *Pax8-Cre; ASPSCR1-TFE3^LSL/+^* fetuses (Figure 2B). Kidney tubules were expanded by large, atypical epithelioid cells with abundant clear to eosinophilic cytoplasm, large round nuclei, and prominent nucleoli (Figure 2B). IHC studies using a human-specific TFE3 antibody revealed high levels of ASPSCR1-TFE3 in the nucleus of atypical tubular cells (Figure 2C). As in human tRCC, ASPSCR1-TFE3–expressing cells had reduced levels of cytokeratin 18 (CK18), a signature epithelial marker (Figure 2D). Overall, these data suggest that ASPSCR1-TFE3 altered cell fate, disrupting nephrogenesis and glomerular development and thus inducing renal failure and postnatal death.

Somewhat surprisingly, despite the well-established pro-tumorigenic role of ASPSCR1-TFE3, the majority of atypical/dysmorphic cells did not appear to be proliferating. Mitotic figures were infrequent, and Ki-67 was low (Figure 2E). In contrast, there was extensive Ki-67 staining in nephron progenitor and other cells of age-matched control kidneys (Figure 2E and Supplemental Figure 3A). Moreover, Ki-67 staining was readily apparent in peritubular cells surrounding ASPSCR1-TFE3*–*expressing cells (Figure 2E and Supplemental Figure 3B). We considered whether increased proliferation of ASPSCR1-TFE3–expressing cells may be offset by cell death, but apoptotic cells were scarce and cleaved caspase-3 levels were low (Figure 2F).

### Sglt2-Cre; ASPSCR1-TFE3 model induces tRCC indistinguishable from human tRCC.

**Figure 3:**
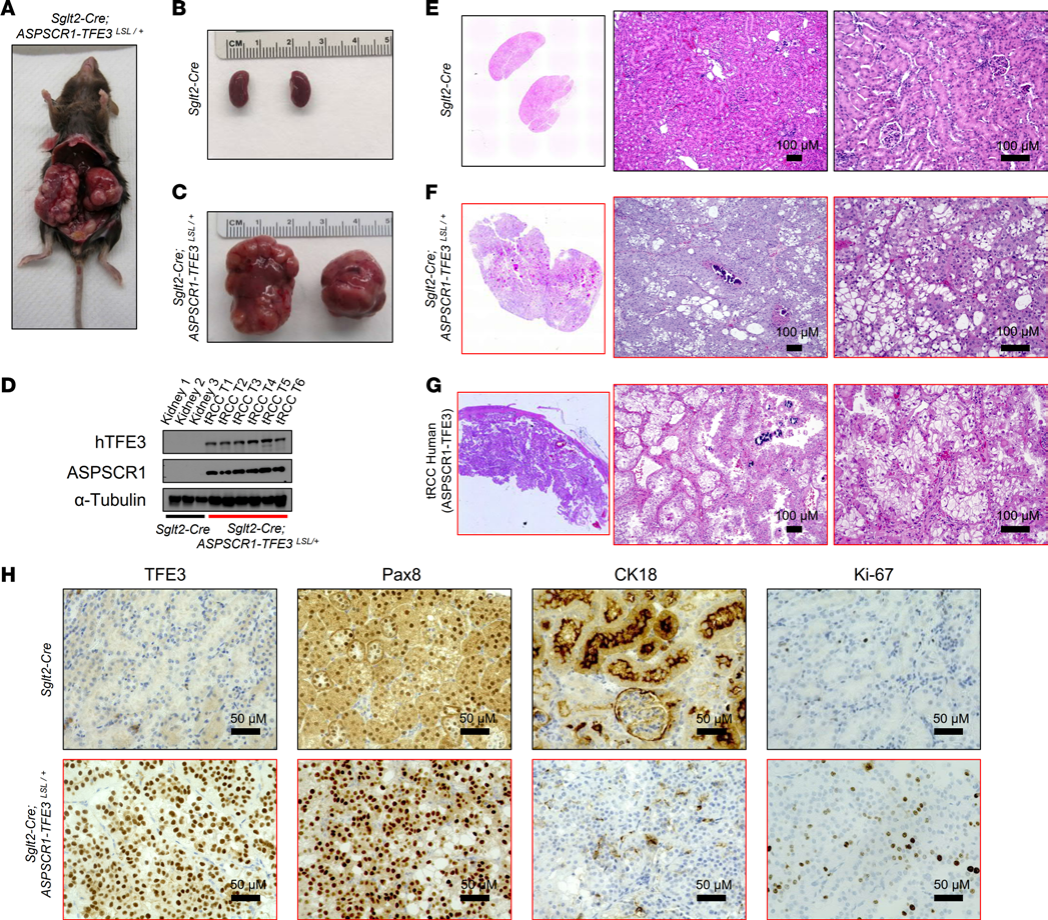
Characterization of *Sglt2-Cre; ASPSCR1-TFE3^LSL/+^* tRCC model. (**A**) Gross anatomical image of 13-month-old *Sglt2-Cre; ASPSCR1-TFE3^LSL/+^* mouse with multiple bilateral renal tumors. (**B** and **C**) Macroscopic images of the kidney from a representative 13-month-old *Sglt2-Cre* (control) (**B**) and an age-matched *Sglt2-Cre; ASPSCR1-TFE3^LSL/+^* mouse (**C**). (**D**) Western blot analysis of *Sglt2-Cre; ASPSCR1-TFE3^LSL/+^* tRCC tumors and control kidneys (*Sglt2-Cre*) for human TFE3 and ASPSCR1. (**E** and **F**) H&E staining of murine kidney tumor and control kidney. Scale bars: 100 μm. (**G**) H&E images of a human *ASPSCR1-TFE3* fusion tRCC. Scale bars: 100 μm. (**H**) IHC for TFE3 (human-specific antibody), Pax8, CK18, and Ki-67 in *Sglt2-Cre; ASPSCR1-TFE3^LSL/+^* tRCC tumor and control mouse kidney. Scale bars: 50 μm.

Next, we crossed *ASPSCR1-TFE3^LSL/+^* mice with *Sglt2-Cre*–expressing mice. In contrast to *Pax8*, *Sglt2* is expressed after birth and is restricted to proximal convoluted tubules (PCTs) (23, 32, 33). *Sglt2-Cre; ASPSCR1-TFE3^LSL/+^* mice were born at expected Mendelian ratios and survived to adulthood. Physical examination revealed tumor development as early as 4 months, and further characterization was performed by MRI (Supplemental Figure 4A). We observed multiple bilateral tumors that could reach more than 1 cm by 1 year of age. These tumors progressively coalesced, eventually replacing the entire kidney (Figure 3, A–C). While most masses were solid, occasionally they formed fluid-filled cysts. Using antibodies against both ASPSCR1 and human TFE3, we documented expression of the fusion protein in tumor lysates (Figure 3D). Histological examination showed tumors with a nested architecture composed of polygonal cells with prominent, well-demarcated, cell borders and abundant clear to granular eosinophilic cytoplasm (Figure 3, E and F). In addition, scattered psammoma bodies (calcifications) were observed in the stroma (Figure 3F). These histological features closely resemble those of human ASPSCR1-TFE3 tRCC (Figure 3G). Also akin to human ASPSCR1-TFE3 tRCC, there was strong and diffuse nuclear TFE3, PAX8 was prominent, and membranous CK18 levels were reduced. While not extensive, Ki-67 staining was consistent with active tumor cell proliferation (Figure 3H).

To further characterize the tRCC tumors, we performed transmission electron microscopy (TEM). Compared with controls, tumor cells showed voluminous cytoplasm, large nuclei, and poorly formed or absent microvilli (Supplemental Figure 4, B and C). At higher magnification, tumor cells were characterized by (a) prominent nuclear membrane irregularities (Supplemental Figure 4, D and E); (b) abnormal mitochondria with significant variation in size and shape (Supplemental Figure 4E); and (c) intracellular glycogen accumulation (Supplemental Figure 4F). Glycogen granules were confirmed by periodic acid–Schiff staining (Supplemental Figure 4, G–I) and, in some cells, were of such prominence as to appear to displace organelles to the periphery (Supplemental Figure 4F). The accumulation of glycogen is in keeping with a body of literature implicating TFE3 in metabolism, and similar findings have been observed with TFE3 overexpression in muscle (34). Thus, conventional histological and TEM studies show that *Sglt2*-*Cre;* *ASPSCR1-TFE3^LSL/+^* induces tRCC tumors that faithfully reproduce the architecture, cell morphology, protein markers, and stroma of human tRCC.

### Sglt2-Cre; ASPSCR1-TFE3 GEMMs develop other tumors, including ASPS.

**Figure 4:**
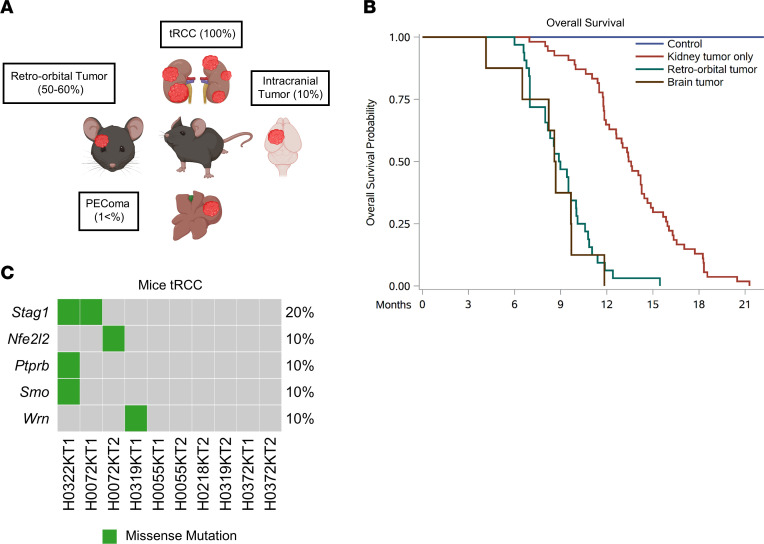
*Sglt2-Cre; ASPSCR1-TFE3^LSL/+^* tumors and tRCC mutational landscape. (**A**) Illustration highlighting tumors in *Sglt2-Cre; ASPSCR1-TFE3^LSL/+^* mice. (**B**) Kaplan-Meier survival analyses of *Sglt2-Cre; ASPSCR1-TFE3^LSL/+^* mice that exclusively developed kidney tumors (*n* = 54) or additional retro-orbital tumors (*n* = 32) or brain tumors (*n* = 8) compared with *Sglt2-Cre* control mice (*n* = 10). (**C**) Oncoprint representation of murine tRCC with somatically mutated genes (COSMIC).

While Sglt2 is thought to be expressed primarily in PCTs, we unexpectedly found that *Sglt2-Cre; ASPSCR1-TFE3^LSL/+^* mice developed tumors in anatomical regions other than the kidney (Figure 4A). Specifically, we observed tumors in the retro-orbital area (50%–60% of mice; Supplemental Figure 5), brain (10%; Supplemental Figure 6, A–I), and liver (<1%; Supplemental Figure 6, J–L). We considered whether these tumors may be metastases, but did not observe metastases in more common destinations such as the lungs, and the tumors were negative for Pax8, a kidney-lineage transcription factor that is usually preserved in metastases (35–37) (Supplemental Figure 5I and Supplemental 6, H and L). Notably, the morphology of the retro-orbital/brain tumors was akin to that of human ASPS (38–41) (Supplemental Figure 5 and Supplemental Figure 6, A–I). In addition, we also observed a liver tumor that, based on morphology and location, may correspond to a human PEComa (Supplemental Figure 6, J–L). Thus, *Sglt2-Cre; ASPSCR1-TFE3^LSL/+^* mice may also serve as a model for other *ASPSCR1-TFE3*–induced tumors such as ASPS and PEComa.

Kaplan-Meier analyses for *Sglt2-Cre; ASPSCR1-TFE3^LSL/+^* mice that exclusively developed kidney tumors showed a median survival of 13.5 months (*P <* 0.0001) (Figure 4B and Table 1). The reduced survival of these mice is likely due to renal failure from tumor outgrowth of the renal parenchyma. Mice that in addition to kidney tumors also developed retro-orbital or brain tumors had a shorter median survival (<9 months; Figure 4B and Table 1). The shorter survival of mice with retro-orbital and brain tumors likely reflects complications associated with intracranial tumor growth.

### Mutation analyses of ASPSCR1-TFE3 mouse tumors.

We performed WES of 10 kidney tumors from 6 *Sglt2-Cre; ASPSCR1-TFE3^LSL/+^* mice (along with corresponding normal samples) (Supplemental Table 5). Mutation analysis implicated a handful of COSMIC genes (Figure 4C and Supplemental Table 6). While none of the genes overlapped with our tRCC human cohort (Figure 4C and Figure 1G), 2 genes were previously reported to be mutated in human tRCC: *Wrn* and *Smo* (29, 42). We expanded these analyses to 4 retro-orbital and 3 brain tumors from the same mice (Supplemental Table 5). We identified 4 genes using stringent criteria, and while none were mutated in more than 1 tumor, interestingly, 2 genes were previously shown to be mutated in human tRCC: *Blm* and *Lrp1b* (29). Parenthetically, the mutation profiles showed that, as previously conjectured, these tumors were not metastases, but rather independent primary tumors (Supplemental Figure 7 and Supplemental Tables 6 and 7).

### Transcriptomic analyses of murine ASPSCR1-TFE3–driven lesions.

**Figure 5:**
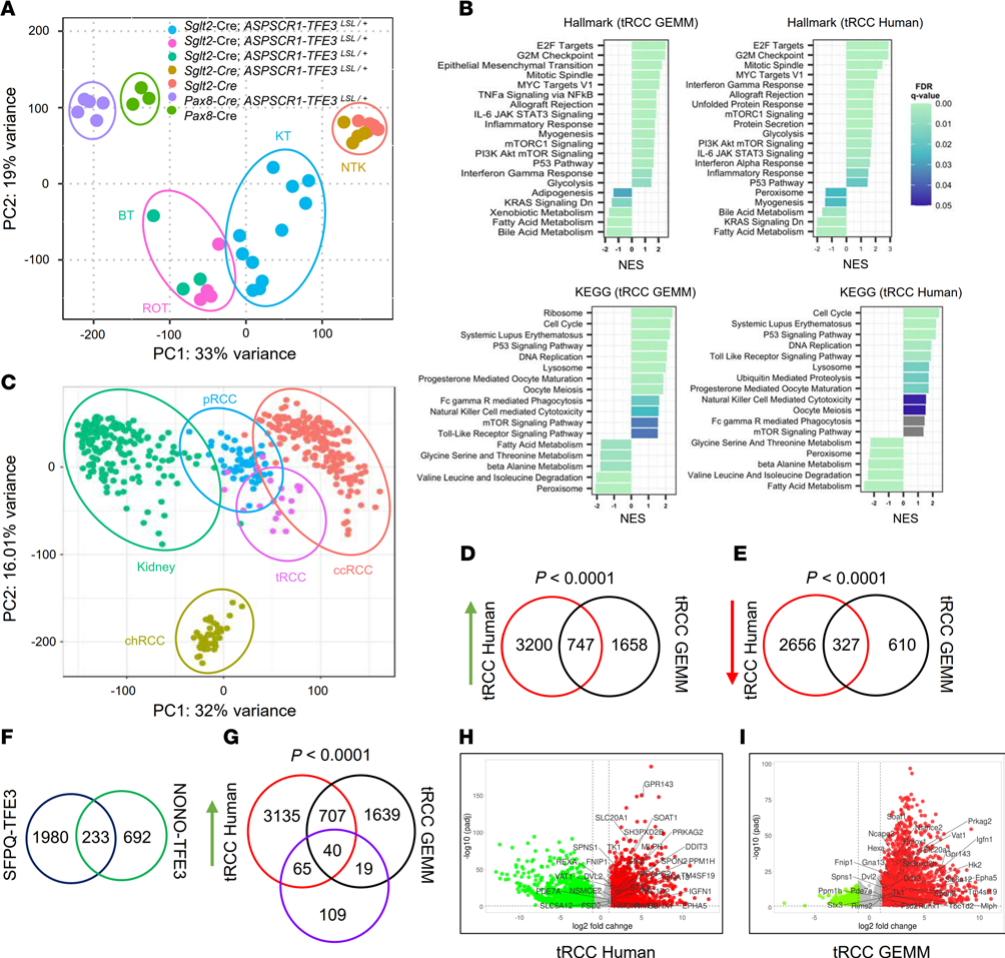
Comparative transcriptomic analyses of murine and human tRCC. (**A**) Principal component analysis (PCA) representation of normalized gene expression read counts of kidney tumors (KT), retro-orbital tumors (ROT), intracranial brain tumors (BT), and non-tumor kidney (NTK) from *Sglt2-Cre; ASPSCR1-TFE3^LSL/+^* mice (or adult Sglt2-Cre kidney controls) as well as deformed kidneys from *Pax8-Cre; ASPSCR1-TFE3^LSL/+^* fetuses and fetal kidney controls (Pax8-Cre). (**B**) GSEA for Hallmark or KEGG gene signatures exhibiting top upregulated and downregulated pathways enriched in murine and human tRCC. NES, normalized enrichment score; FDR, false discovery rate. (**C**) PCA plot representation of normalized gene expression read counts for the UTSW pan-RCC cohort — tRCC (*n* = 19), clear cell RCC (ccRCC) (*n* = 193), papillary RCC (pRCC) (*n* = 55), and chromophobe RCC (chRCC) (*n* = 43) — and tumor-matched normal kidney (*n* = 179). (**D**) Venn diagram of significantly upregulated genes in human and murine tRCC. (**E**) Venn diagram of significantly downregulated genes in human and murine tRCC. (**F**) Venn diagram of SFPQ-TFE3 and NONO-TFE3 direct target genes from 2 independent ChIP-Seq studies. (**G**) Venn diagram of significantly upregulated genes in human/mouse tRCC and their interaction with shared (SFPQ-TFE3 and NONO-TFE3) direct target genes. Hypergeometric tests were carried out to test the overlap between gene expression signatures (**D**, **E**, and **G**). (**H** and **I**) Volcano plots of differentially expressed genes in human (**H**) and murine (**I**) tRCC. Green, downregulated genes; red, upregulated genes; gray, unchanged genes. Putative direct targets of MiT/TFE fusion protein are marked.

We performed transcriptomic analyses for 11 murine tRCC tumors (and 4 adjacent matched normal kidney samples) from 6 mice (Supplemental Tables 5 and 8 and Supplemental Figure 8A). Seeking to understand the relationship between tRCC and the dysmorphic process in *Pax8-Cre; ASPSCR1-TFE3^LSL/+^* fetuses, we also evaluated kidneys from 5 fetuses (as well as from 3 *Pax8-Cre* controls; Supplemental Tables 5 and 9). Principal component analysis (PCA) revealed that the dysmorphic *ASPSCR1-TFE3* fetal kidneys clustered in proximity to age-matched controls and away from tRCC tumors (and adult kidney controls), likely indicating dominance of developmental stage over other *ASPSCR1-TFE3*–related programs (Figure 5A). Gene set enrichment analysis (GSEA) of Hallmark and Kyoto Encyclopedia of Genes and Genomes (KEGG) annotated pathways revealed similarities and differences between dysmorphic kidneys and tRCC (Figure 5B and Supplemental Figure 8B). Specifically, mTOR, epithelial-mesenchymal transition (EMT), and inflammation (inflammatory response, IL-6/JAK/STAT3, TLR, TNF-α/NF-κB, allograft rejection) characterized both dysmorphic kidneys and tRCC. However, tRCC differed from the dysmorphic kidneys by the induction of pathways related to cell proliferation (E2F, Myc, cell cycle, DNA replication, mitotic spindle, etc.) (Figure 5B and Supplemental Figure 8B).

We extended these RNA-Seq analyses to 4 retro-orbital and 3 brain tumors from the same mice (Supplemental Tables 5 and 10 and Supplemental Figure 8A). PCA revealed that retro-orbital and brain tumors clustered together and in proximity to tRCC (Figure 5A). There were substantial similarities between retro-orbital/brain tumors and tRCC, including EMT, lysosome, inflammation (inflammatory response, TNF-α/NF-κB, allograft rejection), and cell proliferation (E2F, Myc, cell cycle, DNA replication, mitotic spindle) (Figure 5B and Supplemental Figure 8B). Overall, these results show that ASPS and tRCC exhibit not only similar morphology, but also gene expression.

### Comparative transcriptomic analyses of human and murine tRCC.

We performed comparative transcriptomic analyses between murine tRCC and human tRCC. For these studies, we used 19 frozen samples from 14 tRCC patients (Supplemental Table 2). First, we contextualized human tRCC gene expression by comparison with a UTSW pan-RCC cohort (193 ccRCC samples from 120 patients; 55 papillary RCC samples from 51 patients; 43 chromophobe RCC samples from 43 patients; as well as 179 normal kidney samples from 178 patients) (7, 26, 27) (Supplemental Table 11 and Supplemental Figure 8C). PCA showed that tRCC samples clustered largely together and away from other subtypes (Figure 5C). Differentially expressed gene (DEG) analysis between human tRCC and normal kidney tissues identified 3,947 overexpressed (log_2_ fold change ≥ 1, adjusted *P* value ≤ 0.05) and 2,983 downregulated (log_2_ fold change ≤ –1, adjusted *P* value ≤ 0.05) genes (Supplemental Table 11 and Figure 5, D and E).

GSEA of human and murine tRCC showed convergence in canonical pathways including lysosome, mTORC1, and proliferative pathways (cell cycle, DNA replication, mitotic spindle) (see also Supplemental Tables 12 and 13). E2F and Myc were induced in both murine and human tRCC and may contribute to increased cell proliferation (Figure 5B). In addition, inflammatory pathways were also prominent, including IFN-γ response, IL-6/JAK/STAT3, systemic lupus erythematosus, TLR signaling, immunoglobulin-mediated phagocytosis, NK cell–mediated cytotoxicity, and allograft rejection.

To more rigorously evaluate the convergence between human and murine tRCC, we focused on murine tRCC genes with human orthologs. We identified 2,405 upregulated and 937 downregulated genes (in comparison with normal kidney samples) (Figure 5, D and E, and Supplemental Table 8). When human and murine DEG analyses were integrated, there were 747 upregulated and 327 downregulated genes that were shared (Figure 5, D and E). Thus, approximately one-third of the genes induced (and downregulated) in murine tRCC were similarly differentially expressed in human tRCC. The probability of observing this amount of overlap (or one more extreme) was statistically significant for both upregulated and downregulated genes (hypergeometric *P* < 0.0001). This convergence illustrates how the mouse model recapitulates the human disease and lays the foundation for comparative analyses to identify core tumorigenic pathways and processes (Supplemental Tables 12 and 13).

We thought to leverage these data to identify novel direct targets of MiT/TFE gene fusions and focused on the intersected genes (between human and mouse tRCC). We performed integrative analyses with previously reported ChIP-Seq results from tRCC lines with *SFPQ-TFE3* (20) and *NONO-TFE3* (43) (Figure 5F). Among 747 overexpressed genes shared by human/mouse tRCC and 233 shared genes across the 2 independent ChIP-Seq studies (Figure 5, D and F), we identified 40 intersecting genes (Figure 5G). Given 19,343 genes shared between human and mouse data sets, the probability of having 40 genes overlap at random between groups of the stated size is *P* < 0.0001. Among the 40 genes there were known MiT/TFE targets such as *HIF1A*, *ANGPTL2*, *BHLHE41*, *CLCN7*, *GPNMB*, *RRAGD*, *SQSTM1*, and *TSPAN10* (21, 30, 44, 45) as well as multiple novel putative targets including *RRAGC*, *FNIP1*, *PRKAG2*, *HK2*, *HMOX1*, *EPHA5*, and *RUNX1* (Figure 5, H and I, and Supplemental Table 14).

### Simultaneous induction of mTORC1 and autophagy programs in tRCC.

**Figure 6:**
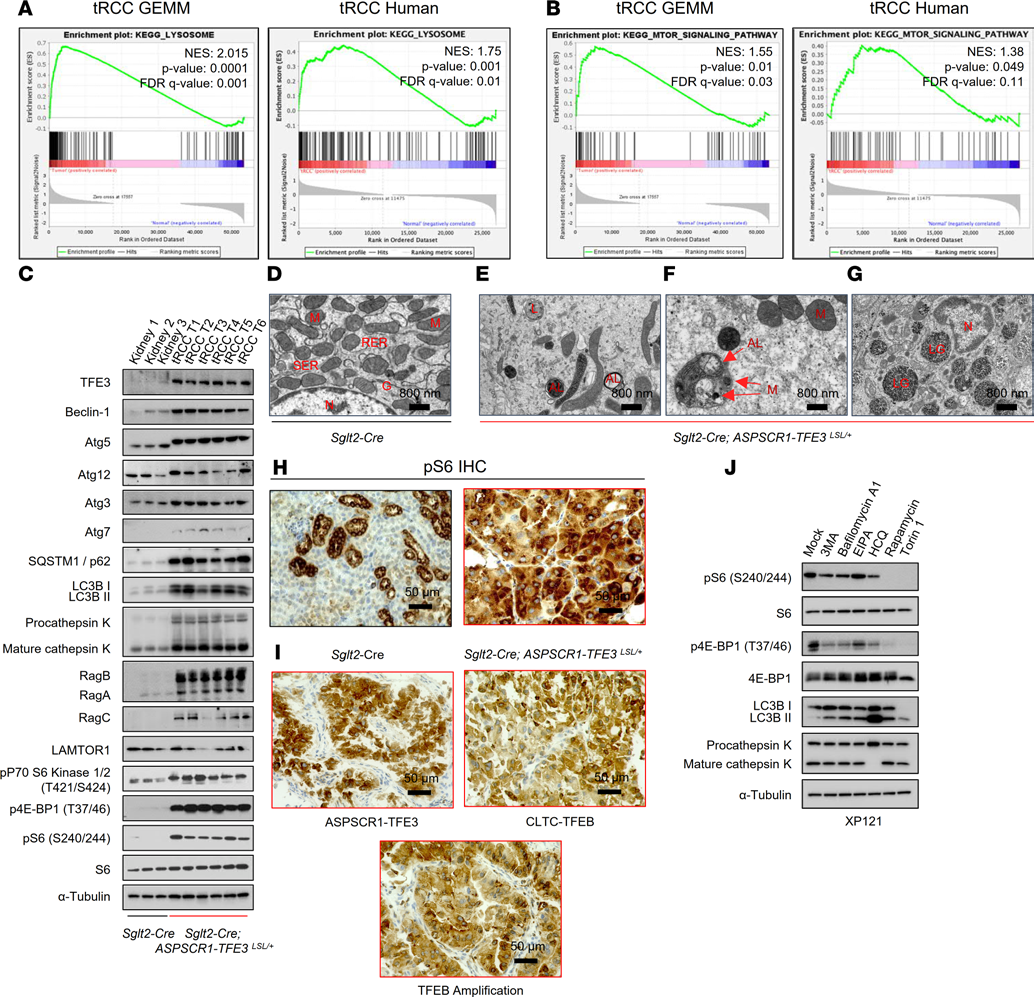
Simultaneous activation of mTORC1 and autophagy-lysosome pathways in tRCC. (**A** and **B**) GSEA plots for lysosome (**A**) and mTOR signaling pathway (**B**) in murine and human tRCC. NES, normalized enrichment score; FDR, false discovery rate. (**C**) Western blot analyses of murine tRCC (*Sglt2-Cre; ASPSCR1-TFE3^LSL/+^*) and control kidneys (*Sglt2-Cre*). Assembly from membranes of different gels run with the same protein lysate. (**D**–**G**) Representative transmission electron micrographs of *Sglt2-Cre* kidney (control) (**D**) and tRCC from *Sglt2-Cre; ASPSCR1-TFE3^LSL/+^* mice showing atypical mitochondria and abundant lysosome/autolysosome (**E**), engulfed organelles (**F**), and intra-lysosomal glycogen (**G**). AL, autolysosome; G, Golgi; L, lysosome; LG, lysosomal glycogen; M, mitochondria; N, nucleus; RER, rough endoplasmic reticulum; SER, smooth endoplasmic reticulum. Scale bars: 800 nm. (**H**) IHC for phospho-S6 of murine tRCC model and control. Scale bars: 50 μm. (**I**) IHC for phospho-S6 of human tRCC (representative cases shown with *TFE3* gene fusion, *TFEB* gene fusion, and *TFEB* amplification). Scale bars: 50 μm. (**J**) Western blot analysis of XP121 cells treated with 3MA (5 mM), bafilomycin-A1 (1 nM), EIPA (50 μM), hydroxychloroquine (HCQ; 25 μM), rapamycin (100 nM), and Torin 1 (250 nM) for 24 hours.

An apparent paradox of tRCC, which was also observed in our GSEA, is the simultaneous induction of catabolic (lysosome) and anabolic (mTORC1) programs (Figure 6, A and B). GSEA revealed an induction of lysosomal hydrolytic enzymes and transmembrane and transport proteins, as well as components of the amino acid sensing machinery of mTORC1, such as Rag GTPases and FNIP1. To evaluate this further, we performed Western blot and IHC studies of autophagy-lysosome and mTORC1 signaling proteins. We analyzed 6 tRCC murine tumors and 3 normal kidney samples. Western blotting revealed an increase in the expression of proteins associated with autophagy (Atg3, Atg5, Atg7, SQSTM1/p62, and LC3B) and the lysosome (cathepsin K) (Figure 6C). Moreover, high ratios of LC3II/I and mature/procathepsin K suggested ongoing autophagy (Figure 6C). Conversely, we also observed an induction of Rag GTPases, including RagB and RagC, and markers of mTORC1 activation (phospho–4E-BP1, phospho–p70 S6K, and phospho-S6) (Figure 6C). The increased expression of lysosomal proteins corresponded to an abundance of lysosomes and associated organelles (autophagosomes and autolysosomes) detected by TEM (Figure 6, D–F). We also observed engulfment of organelles (including mitochondria) as well as of glycogen granules (Figure 6, F and G). These results expand on similar findings in human tRCC (46). Regarding mTORC1, phospho-S6 was prominent in both murine and human tRCC (Figure 6, H and I), and the levels were higher than in ccRCC (Table 2). Overall, these results support the notion that in both human and murine tRCC, autophagy and mTORC1-dependent anabolic pathways are activated.

To explore the interplay between autophagy-lysosome and mTORC1, we evaluated the impact of drugs that interfere with autophagy-lysosome using the *PRCC-TFE3*–expressing cell line we generated (XP121). We tested drugs that interfere with autophagy initiation and maturation, autophagy-lysosome fusion, and lysosome acidification (3MA, bafilomycin-A1, and hydroxychloroquine) (Figure 6J). As controls, we evaluated mTORC1 inhibitors (rapamycin and Torin 1) and EIPA [5-(N-Ethyl-N-isopropyl)-Amiloride], an inhibitor of micropinocytosis. Lysosome inhibitors, but not EIPA, led to a modest downregulation in mTORC1, showing that these pathways are interconnected.

### Targeting growth factor signaling pathways in tRCC.

**Figure 7:**
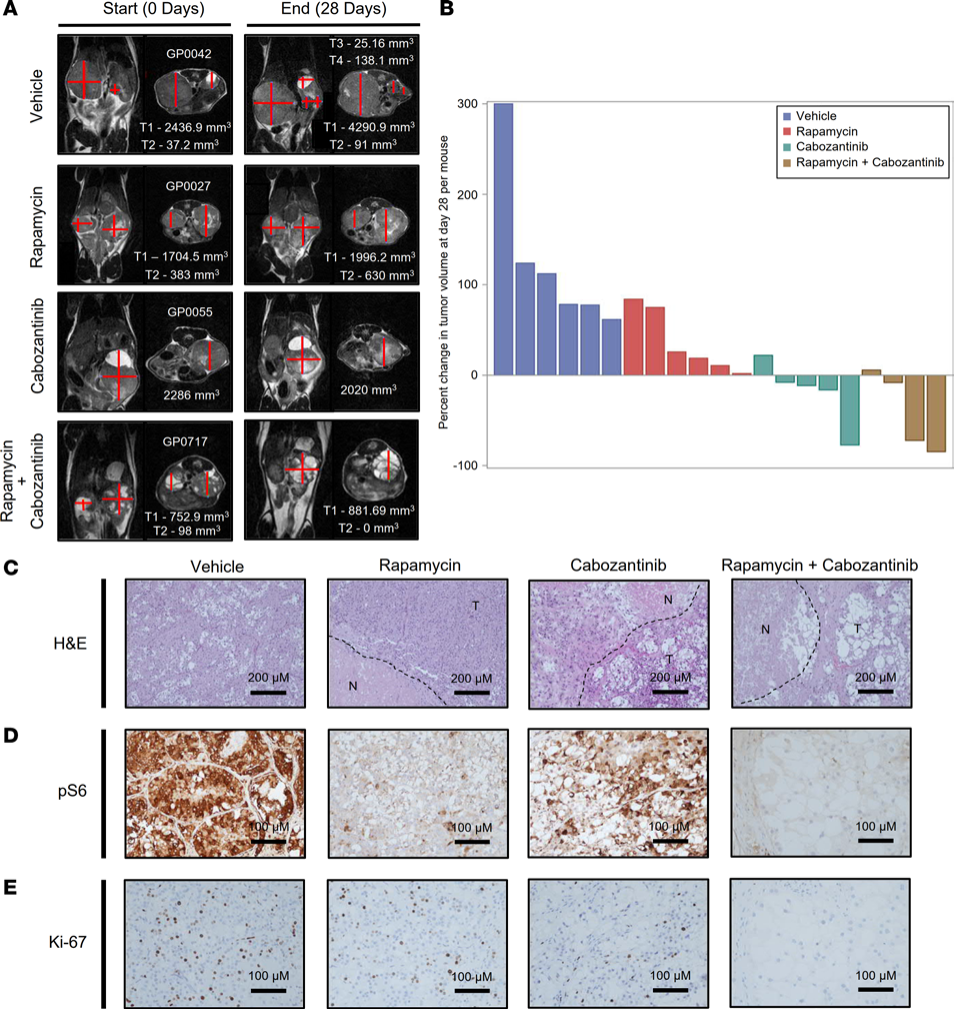
Inhibition of tRCC growth by cabozantinib and rapamycin. (**A**) Representative MRI images of *Sglt2-Cre; ASPSCR1-TFE3^LSL/+^* mice with kidney tumor volume measurements (see Methods) at baseline and end of the trial. (**B**) Waterfall plot with percentage change in overall kidney tumor burden per mouse. (**C**–**E**) Representative H&E and IHC (phospho-S6 and Ki-67) at the end of drug trials. N, necrosis; T, tumor. Scale bars: 200 μm (**C**); 100 μm (**D** and **E**).

To evaluate the importance of mTORC1 activation in tRCC tumor development, we treated *Sglt2-Cre; ASPSCR1-TFE3^LSL/+^* mice with rapamycin using a dosing regimen we previously showed mimicked human exposures (27). Rapamycin inhibited mTORC1 in tumors and decreased tumor growth (*P* = 0.013) (Figure 7, A and B, Tables 3 and 4, and Supplemental Figure 9, A and B). However, despite mTORC1 inhibition (Figure 7, C–E), the antiproliferative effect was modest. Next, we investigated other growth factor signaling pathways. The hepatocyte growth factor receptor MET is induced by TFE3 fusion proteins, which can directly transactivate the gene (47). Consistent with this notion, *MET* was induced in our human tRCC cohort (as well as another cohort we examined) and was among the putative direct target genes (Supplemental Figure 10, A and B). However, *Met* was not induced in murine tRCC (Supplemental Figure 10C). In contrast, murine tRCC induced *Ret* (Supplemental Figure 10, D–G), which is consistent with a recent study (21). Interestingly, MET and RET kinases functionally interact, and *MET* amplification has been shown to cause resistance to RET inhibitors (48). To assess the impact of MET/RET, we evaluated the tyrosine kinase inhibitor cabozantinib, which has activity against both kinases. We performed pharmacokinetic (PK) analyses of cabozantinib in mice to identify a regimen that reproduces human exposures. PK studies (Table 5) and subsequent modeling (Phoenix WinNonlin PK Model 4, Certara Corp.) suggested an oral dose of 5 mg/kg twice daily to approximate steady-state exposures in humans on conventional doses of 60 mg/d (49, 50). Treatment of tumor-bearing *Sglt2-Cre; ASPSCR1-TFE3^LSL/+^* mice with cabozantinib using this dosing regimen significantly inhibited tumor growth (*P <* 0.001). However, the effect was not synergistic with rapamycin (Figure 7, A–E, Tables 3 and 4, and Supplemental Figure 9, A and B).

## Discussion

In this study, we present integrated analyses of a human tRCC cohort and a novel GEMM, yielding insights into key biological pathways underlying tRCC development.

Advances in our understanding of the molecular pathogenesis of tRCC have been hampered by a lack of mouse models reproducing the aggressiveness of the human disease. Previous models involving *PRCC-TFE3* (21) and *TFEB* overexpression (22) developed cysts as well as microscopic/small tumors. In contrast, *Sglt2-Cre; ASPSCR1-TFE3^LSL/+^* mice develop large, macroscopic tumors with complete penetrance and short latency. These tumors are so aggressive that they coalesce over time, replacing the kidney, leading to a premature death (median survival, 13 months). There are several reasons for the differences across the models, including the type of translocation (*ASPSCR1-TFE3* may be associated with particularly aggressive disease) (30) and, perhaps most significantly, the Cre driver. Previous studies used a cadherin 16 (Cdh16/KSP) driver, but as we show, different Cre drivers can result in different phenotypes. *Sglt2-Cre* drives tumors that reproduce the features of human tRCC not only in its aggressiveness, but also at multiple other levels, including histologically (tumor architecture, cytology, and stromal features), ultrastructurally (expansion of lysosomes and autophagosomes), immunohistochemically (downregulation of epithelial markers and preservation of Pax8), and functionally (activation of cell cycle, autophagy-lysosome, and mTORC1 pathways). There is also extensive convergence in gene expression analyses.

Notably, Sglt2-Cre–driven *ASPSCR1-TFE3* induced not only tRCC, but also retro-orbital and intracranial ASPS. While it could be argued, based on their resemblance, that these tumors were tRCC metastases, both lineage studies and WES analyses show that they are independent primary tumors. Interestingly, ASPS in pediatric and adolescent patients preferentially occurs in the head and neck region, especially the orbit and tongue (39, 51, 52). In addition, ASPS tends to develop more often in females (38–41), and this was also the case in *Sglt2-Cre; ASPSCR1-TFE3^LSL/+^* mice. ASPS and tRCC showed substantial overlap at multiple levels. Histologically, tumor cells had a similar appearance, and by gene expression, these tumors showed activation of some of the same pathways, including EMT, lysosome, cell proliferation (E2F, Myc, cell cycle, DNA replication, mitotic spindle), and inflammation (inflammatory response, TNF-α/NF-κB, allograft rejection). These data suggest similar molecular pathogenesis between ASPS and tRCC. Furthermore, ASPS tumors showed mutations in *Blm* and *Lrp1*, which were previously found to be mutated in human tRCC (29). While we cannot exclude leakiness of the Cre driver, careful analyses of the human protein atlas show occasional Sglt2-positive cells in the brain and in the eye, which could give rise to ASPS. This convergence on an Sglt2-expressing cell for both ASPS and tRCC is unexpected and notable.

We also observed a tumor in the liver, which was most suggestive of a liver PEComa. In humans, PEComas generally have mutations in the *TSC1*/*TSC2* genes, but MiT/TFE translocations have been observed when those are absent (16, 17), suggesting that the pathways may converge, which is consistent with our earlier pioneering studies (18). Thus, these GEMMs serve to model not only human tRCC but also ASPS and possibly PEComa.

Beyond the *Sglt2-Cre; ASPSCR1-TFE3^LSL/+^* model, we expand the number of available tRCC models through the characterization of several TGs in our collection (26). These TG models reproduce the features of patient tumors, which is perhaps best shown by their pairing together in unsupervised analyses of gene expression. To our knowledge, TG models have been reported only for *SFPQ-TFE3* (20). In addition to a second *SFPQ-TFE3* model, we present models of *ASPSCR1-TFE3*, *PRCC-TFE3*, and *RBM10-TFE3* tRCC. We have also developed a model for a *TFEB* translocation, *MALAT1-TFEB*, where TG lines were created from both the primary tumor and a lymph node metastasis. While these models require an immunocompromised host, TGs reproduce the mutations, gene expression, and treatment responsiveness of patient tumors and therefore have broad utility (26, 27).

Interestingly, ASPSCR1-TFE3 expression gave rise to 2 different phenotypes in the mouse depending on the driver. In the *Sglt2* lineage, tumors formed and were quite aggressive. In contrast, *ASPSCR1-TFE3* expression in the nephron progenitor (Pax8) lineage disrupted nephrogenesis, abrogating the development of glomeruli without accompanying expansile lesions. *ASPSCR1-TFE3*–expressing cells were similar in both lineages with respect to their morphology, loss of epithelial markers, EMT, and mTORC1 activation. Interestingly, morphological changes were observed as early as E13, suggesting that cell fate transitions occur rapidly. GSEA showed induction of cell proliferation programs, as well as Myc and E2F, in tumors, but not in the dysmorphic kidneys. These data suggest an uncoupling of *ASPSCR1-TFE3* cell fate and proliferation programs depending on cell context. Findings in the Pax8 model are consistent with observations in cell lines where MiT/TFE overexpression in certain contexts causes senescence (53). However, whether *ASPSCR1-TFE3* expression in the nephron progenitor (Pax8) lineage induces senescence remains to be determined. Furthermore, since the respective controls for Sglt2-Cre and Pax8-Cre mice differed (adult vs. embryonic kidney), we cannot exclude that comparably lower rates of cell proliferation in the dysmorphic kidneys are due, at least in part, to proportionally higher proliferation rates in the embryonic kidney compared with the adult.

In contrast, as we showed previously, Pax8-Cre can induce different types of ccRCC. Inactivation of *Vhl* together with *Pbrm1* in the Pax8 lineage induces low-grade ccRCC, while disruption of *Vhl* and *Bap1* induces distinctive high-grade tumors (23). In addition, low-grade *Vhl*/*Pbrm1* tumors can transform into higher-grade tumors by deleting *Tsc1* and activating mTORC1 (23). Thus, while multiple ccRCC oncogenotypes can be induced by Pax8-Cre, *Pax8-Cre; ASPSCR1-TFE3^LSL/+^* mice did not develop expansile lesions. It remains to be determined, however, whether failure to develop expansile lesions is due to the premature demise of the mice, in particular since tRCC tumors in the Sglt2 lineage (as in humans) were Pax8 positive. It is also noteworthy that while *Vhl*, *Pbrm1*, and *Bap1* are all essential genes and their knockout results in embryonic death (54–56), their conditional loss in the Pax8 lineage was tolerated while *ASPSCR1-TFE3* expression in the same lineage was not.

Conversely, Sglt2-Cre–driven loss of *Vhl*/*Pbrm1* and *Vhl*/*Bap1* did not induce ccRCC (23). This is particularly striking given that Sglt2 is expressed in PCT cells, where it encodes for a sodium-glucose cotransporter that is responsible for 90% of glucose reabsorption, which occurs in the PCT (57). Thus, even though (a) the Sglt2 lineage is capable of transformation, and (b) ccRCC is thought to arise from PCT cells, the finding that Sglt2-expressing PCT cells are resistant to transformation by loss of *Vhl*/*Pbrm1* or *Vhl*/*Bap1* shows that (a) the cells of origin of tRCC and ccRCC are different, and (b) ccRCC does not arise from Sglt2-expressing PCT cells. In keeping with this notion, our previous studies suggest that at least for some ccRCC, the cell of origin is not in the PCT, but rather a rare stem cell found in the parietal layer of the Bowman capsule (23). We speculate that whereas *ASPSCR1-TFE3* can transform differentiated PCT cells, transformation by loss of *Vhl*/*Pbrm1* or *Vhl*/*Bap1* requires a less differentiated cellular state.

Given how faithfully the *Sglt2-Cre; ASPSCR1-TFE3^LSL/+^* model reproduces human tRCC, we sought to exploit the model to obtain insight into human tRCC biology. We thought we might leverage the greater simplicity and more controlled environment of murine tRCC to identify core tumorigenic processes. For these experiments, we performed comparative studies between murine and human tRCC. Gene expression analyses revealed significant convergence in canonical pathways (cell cycle, lysosome, and mTORC1) as well as less established pathways such as Myc, E2F, and inflammation (IFN-γ response, IL-6/JAK/STAT3, systemic lupus erythematosus, TLR signaling, immunoglobulin-mediated phagocytosis, NK cell–mediated cytotoxicity, and allograft rejection). Our studies also revealed putative direct MiT/TFE fusion protein targets including the mTORC1 regulators *RRAGC* and *FNIP1*, which encode proteins involved in mTORC1 activation by amino acids, as well as several genes implicated in energy sensing and metabolism such as *PRKAG2*, *HK2*, and *HMOX1*.

We observed simultaneous activation of TFEB/TFE3-mediated autophagy-lysosome biogenesis and mTORC1 pathways in both murine and human tRCC. Concurrent activation of catabolic and anabolic pathways is somewhat paradoxical. Under physiological conditions, mTORC1, a master regulator of cell growth, inhibits TFEB/TFE3 proteins and autophagy (19, 58). This process involves TFEB/TFE3 recruitment to the surface of the lysosome through binding to Rag GTPases and mTORC1 phosphorylation (59, 60). TFEB/TFE3 phosphorylation by mTORC1 results in cytoplasm sequestration in a manner that is, at least in part, dependent on 14-3-3 protein binding (61, 62). There is precedent, however, for simultaneous activation of TFEB/TFE3 and mTORC1 pathways in disease conditions, as we previously reported for the first time in tuberous sclerosis complex (18). Since then, a similar phenomenon has been observed in Birt-Hogg-Dubé syndrome, which is similarly associated with renal tumors (63). However, how mTORC1 is activated is poorly understood. TFE3 fusion proteins appear to induce the expression of RagD (45), which is implicated in mTORC1 activation by amino acids (64–67). Interestingly, we found that RagB, RagC, and FNIP1 were induced in our murine tRCC tumors, suggesting that mTORC1 regulation by TFEB may extend beyond RagD, at least in tRCC. We speculate that several processes interfere with physiological mTORC1-mediated inhibition of TFEB/TFE3 in tRCC. First, both amplifications and translocations lead to substantial overexpression (68), which may be sufficient to activate TFEB/TFE3 (22, 69, 70). Second, most translocations lead to loss of the N-terminus, which has been implicated in binding to Rag GTPases and recruitment to the surface of the lysosome where TFEB/TFE3 becomes phosphorylated by mTORC1 and sequestered away from the nucleus (59, 63). Finally, deletion of the N-terminus in TFE3 also eliminates a phospho-degron implicated in TFE3 degradation (71).

Our gene expression analyses show induction of inflammatory pathways in tRCC, including IFN-γ response, IL-6/JAK/STAT3, TLR signaling, immunoglobulin-mediated phagocytosis, systemic lupus erythematosus, NK cell–mediated cytotoxicity, and allograft rejection. While at face value these inflammatory programs may be taken to reflect infiltrating inflammatory cells, we cannot exclude a cell-autonomous effect, and TFE/MiT proteins have been implicated in the activation of inflammatory pathways (72, 73). Furthermore, while inflammatory/immune cells were observed around tumors, they were rarely found within tumors. Spatial transcriptomic analyses should help dissect the process. Nevertheless, shared inflammatory/immune signatures between murine and human tRCC suggest that the *Sglt2-Cre; ASPSCR1-TFE3^LSL/+^* model could be used for immunotherapy development, and the early development of tumors makes these experiments possible.

An open question is how MiT/TFE fusion proteins induce cell proliferation. Previous studies have implicated the receptor tyrosine kinases MET (47) and RET (21). MET and RET activate similar signal transduction pathways, and *MET* amplification has been shown to cause resistance to RET inhibitors (48). In our studies, we observed *MET* overexpression in human tRCC and *Ret* overexpression in murine tRCC. A MET/RET antagonist, cabozantinib, showed antitumor activity, but inasmuch as cabozantinib targets other kinases, the relative contribution of MET/RET is unclear. Nevertheless, these data support the evaluation of cabozantinib for tRCC, a concept that is being explored in clinical trials (NCT03685448, ClinicalTrials.gov).

We sought to leverage our *Sglt2-Cre; ASPSCR1-TFE3^LSL/+^* model to provide insight into the molecular genetics of tRCC. Previous studies in humans have shown that tRCC is characterized by a low mutation burden (~0.5 mutations/Mb) (30, 42), and most mutated genes are mutated in just a few tumors. Furthermore, few mutated genes overlap across studies. In fact, to our knowledge only *TP53* and *SMARCA4* have been implicated as possible tRCC driver genes in more than one study (29, 30, 42, 74, 75). In our human cohort we found mutations in *DNMT3A* in 2 tumors, and *DNMT3A* was previously shown to be mutated in another study (29). As for transcriptomic analyses, we hoped that WES of murine tRCC would be similarly informative. Disappointingly, however, we found only 2 genes that were previously mutated in human tRCC, *Wrn* and *Smo* (29, 42). *Wrn* encodes a multifunctional enzyme with helicase and exonuclease activity, which functions as a tumor suppressor. However, given the low prevalence of *WRN* mutations in tRCC (both human and murine), whether it is implicated in tRCC pathogenesis remains to be determined. Overall, multiple studies have shown that tRCC tumors have few, if any, unequivocal mutations in additional driver genes. These results are open to several interpretations. The simplest interpretation is that *ASPSCR1-TFE3* is sufficient for tumor development and no other cooperating events are required. However, not every *ASPSCR1-TFE3*–expressing cell in the Sglt2 lineage appeared to give rise to a tumor. An alternative interpretation is that MiT/TFE translocations cooperate with multiple genes in tumorigenesis without a strong preference except perhaps for *TP53* and *SMARCA4*.

Finally, studies of our institutional cohort serendipitously identified a hitherto unreported translocation, *SFPQ-TFEB*, which expands our previous findings of *TFEB* amplification and *MITF* translocation in tRCC (7). Like the *SFPQ* fusion with *TFE3* (10), we mapped the chromosomal breakpoint downstream of *SFPQ* exon 9. This fused with *TFEB* exon 4, rendering a chimeric protein lacking the first 71 amino acids of TFEB but retaining the bHLH DNA-binding and leucine zipper dimerization domains with strong nuclear accumulation. Interestingly, the translocation coexisted with *TFEB* amplification.

In conclusion, through the generation of several mouse models and integrative genomic studies including a human tRCC cohort, we provide insight into molecular mechanisms of *ASPSCR1-TFE3*–mediated transformation and add to the models available for the research community.

## Methods

### Sex as a biological variable.

We examined both male and female animals, in which we found similar rates of tRCC, but consistent with studies in humans, we observed higher prevalence of ASPS retro-orbital tumors in female mice.

### Genetically engineered mouse models.

*ASPSCR1-TFE3^LSL/+^* mice have a full-length *ASPSCR1-TFE3* type 2 cDNA (ASPSCR1, exons 1–7; TFE3, exons 5–10) preceded by a *loxP*-stop-*loxP* (LSL) cassette in the *Rosa26* locus (31). *ASPSCR1-TFE3^LSL/+^* mice were interbred with mice expressing Cre under the control of kidney lineage–specific promoters, *Pax8* and *Sglt2*. *Sglt2-Cre* mice were a gift from Michel Tauc and Isabelle Rubera (Université Côte d’Azur, Laboratoire PhysioMédecine Moléculaire, Nice, France) (33), and *Pax8-Cre* mice were provided by Meinrad Busslinger (Research Institute of Molecular Pathology, Vienna, Austria) (76). All mice were housed in a barrier facility with sterile laminar flow cages maintained at 22°C, under a 12-hour light/12-hour dark cycle. Animals were provided with unrestricted access to disinfected tap water and commercial food. The phenotype of *Pax8-Cre;* *ASPSCR1-TFE3^LSL/+^* fetuses was characterized by comparison with control littermates (*ASPSCR1-TFE3^LSL/+^* or *Pax8-Cre*) obtained from at least 5 pregnant females. More than 10 *Pax8-Cre;* *ASPSCR1-TFE3^LSL/+^* fetuses were analyzed. For *Sglt2-Cre;* *ASPSCR1-TFE3^LSL/+^*, we analyzed over 100 mice. Genotyping primer sequences are as follows: *ASPSCR1-TFE3^LSL/+^*, LSL-AT3 A, 5′-GTTATCAGTAAGGGAGCTGCAGTGG-3′; LSL-AT3 B, 5′-AAGACCGCGAAGAGTTTGTCCTC-3′; LSL-AT3 C, 5′-GGCGGATCACAAGCAATAATAACC-3′; Sglt2-Cre, Sglt2-Cre A, 5′-AGGCTGAGGAATGTGTTGAGG-3′ (Sglt2 intron 1); Sglt2-Cre B, 5′-CAAACTGGGCTGTCCCAACT-3′ (Sglt2 exon 3); Sglt2-Cre C, 5′-CAGGGTGTTATAAGCAATCCC-3′ (Cre reverse); Pax8-Cre, Pax8-Cre A, 5′-GTACCTAGCCATGCCCTCAC-3′ (Pax8 intron 2); Pax8-Cre B, 5′-TTCGTGCTTACCTGCCAAGG-3′ (Pax8 exon 3); Pax8-Cre C, 5′-CAGGGTGTTATAAGCAATCCC-3′ (Cre reverse).

### Tumorgraft mouse models.

Detailed procedures for tumorgraft (TG) generation have been reported previously (26–28). In brief, approximately 4- to 6-week-old male or female, nonobese diabetic/severe combined immunodeficient NOD.CB17-Prkdc^scid^ (NOD/SCID) mice were implanted with tumor samples from patients orthotopically without additives or disaggregation. Upon reaching approximately 10 mm diameter, TGs were passaged to successive cohorts. The nomenclature scheme was assigned as previously described (26). Briefly, TG lines are given a unique “XPID,” which consists of a prefix “XP” followed by a numerical value. Several XPIDs may be derived from a single patient, if tumors are collected from multiple sites including the primary and metastatic sites. We report here 7 tRCC TGs generated from 6 tRCC patients (Supplemental Table 2). With the exception of XP506 (KC01122), which was derived from ascitic fluid, and XP1187 (KC03023), which was derived from lymph node metastasis, all other cases were derived from the primary tumor. From one of the TGs (KC01017; XP121), we generated a primary cell line using methods we previously described (77).

### Fluorescence in situ hybridization.

FISH for the identification of *TFEB*/*TFE3* alterations using DNA probe sets (Agilent Technologies) that hybridize distal and proximal to the *TFE3* or *TFEB* gene on interphase nuclei from the patient specimens was carried out during routine diagnostic workup either at our institutional CLIA-certified molecular laboratory or elsewhere (Propath or Mayo Clinic laboratories). Approximately 200 non-overlapping interphase nuclei were examined per specimen, and 10 or more cells with an aberrant probe signal pattern were required to call an abnormality.

### Supplemental materials.

Further information can be found in Supplemental Methods.

### Statistics.

To analyze survival, Kaplan-Meier curves with log-rank tests were used. For drug trials, a linear regression was used to test for an association between treatment and change in overall tumor burden per mouse. Mixed-effects modeling was used to test for an association between treatment and change in individual tumor volumes, accounting for the correlation between tumors in the same mouse. For both linear regression and mixed-effects models of the drug trial data, models were built with an interaction term between rapamycin and cabozantinib to evaluate for possible synergy of the treatments. We used a hypergeometric test to analyze whether there was significant overlap in gene expression signatures. A χ^2^ test was used to test for an association between phospho-S6 staining and RCC histology (ccRCC vs. tRCC). All tests were 2-tailed and were performed at the 0.05 significance level using SAS 9.4 (SAS Institute Inc.), unless otherwise noted. Statistical significance for differences in *MET* and *RET* gene expression in normal versus tumor was tested using an unpaired Student’s *t* test (GraphPad Prism).

### Study approval.

Written informed consent was obtained for samples that were prospectively collected and studies were conducted following the University of Texas Southwestern Medical Center Institutional Review Board–approved protocols STU-012011-190, STU-22013-052, and STU-02215-015. Clinicopathological characteristics for the 30 tRCC patients enrolled were abstracted from Kidney Cancer Explorer (KCE), and a deidentified version is made available (Supplemental Tables 1 and 2). A unique KCE identifier (KCEID) was assigned to each patient (Supplemental Table 2). Murine studies were carried out in accordance with and with the approval of UTSW institutional animal care and use committee protocol APN#2015-100932.

### Data availability.

The raw and processed data obtained from bulk RNA-Seq analysis of *Pax8-Cre; ASPSCR1-TFE3^LSL/+^* and *Sglt2-Cre; ASPSCR1-TFE3^LSL/+^* mice were deposited in the NCBI Gene Expression Omnibus database (GEO GSE252047). WES data for mice generated in this study were deposited in the NCBI Sequence Read Archive database under the BioProject identifier PRJNA1053626. Previously unpublished sequencing files from tRCC patients who provided informed consent for release of genomic information are available in the European Genome-Phenome Archive (EGA) under the following IDs: WES, EGAD50000000171; RNA-Seq, EGAD50000000172. EGA identifiers for previously published sequencing data from tRCC patients and TGs are provided in Supplemental Table 2 (7, 26). Specifically, previously reported sequencing data for tRCC patients are accessible with the following IDs: EGAD00001001022, EGAD00001001023 (7), EGAD00001008010 (25); and for TGs: EGAD00001007989 (26). The data values corresponding to the plotted points in the graphs presented can be found in the Supporting Data Values file.

## Author contributions

GP, PK, and JB conceptualized the study. GP, HY, PK, and JB acquired tRCC models and performed phenotype characterization. VTT, GP, PK, and JB acquired and validated tRCC TG models. AM, GP, ORT, YW, and TW performed genomic analyses. AC performed statistical analysis. GP, AC, VTT, and NSW performed drug trials. JM, DC, GP, and PK performed immunohistochemistry. DR and GP performed electron microscopy. QND and IP performed MRI. GP, AM, AC, VTT, DR, QND, IP, YW, TW, HZ, JG, NSW, PK, and JB performed data curation. GP wrote the original draft of the manuscript. GP, AM, AC, JM, VTT, QND, ORT, RB, HZ, JG, KBJ, TJC, ZM, SD, MCRK, NSW, IP, TW, DR, PK, and JB reviewed and edited the manuscript. JG, KBJ, TJC, ZM, SD, IP, DR, PK, and JB provided resources. TW, IP, PK, and JB supervised the study. JB acquired funding.

## Supplementary Material

Supplemental data

Unedited blot and gel images

Supplemental tables 1-14

Supporting data values

## Figures and Tables

**Table 4:**
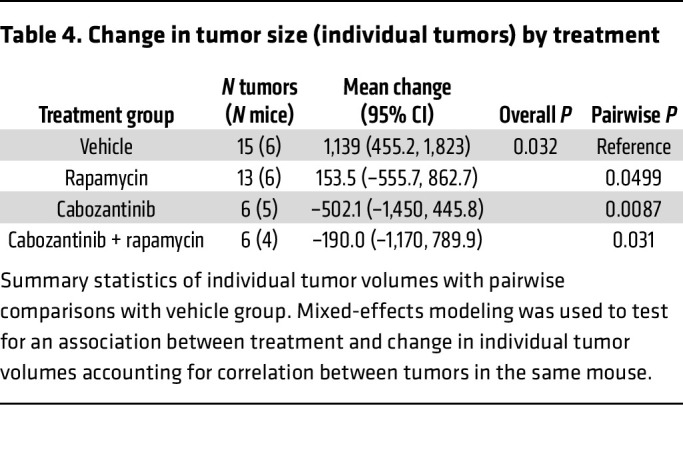
Change in tumor size (individual tumors) by treatment

**Table 3:**
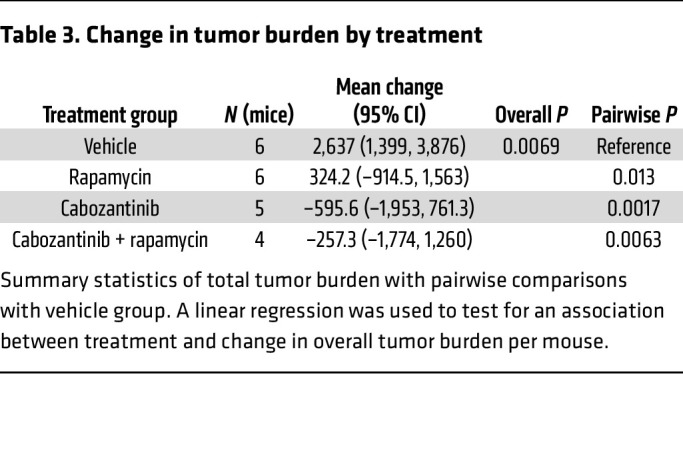
Change in tumor burden by treatment

**Table 2:**
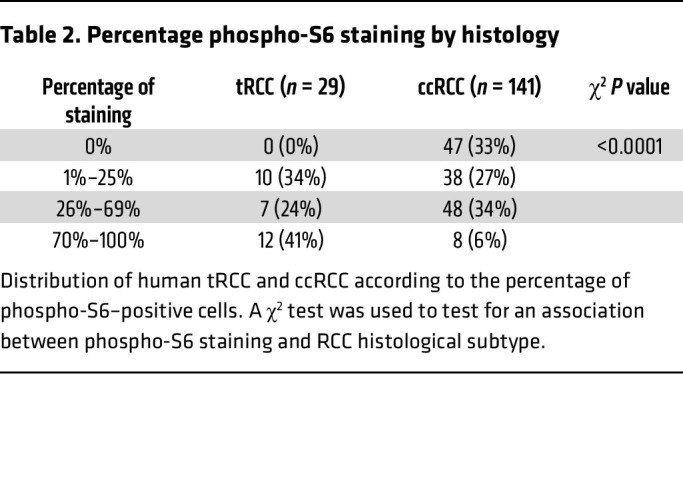
Percentage phospho-S6 staining by histology

**Table 1:**
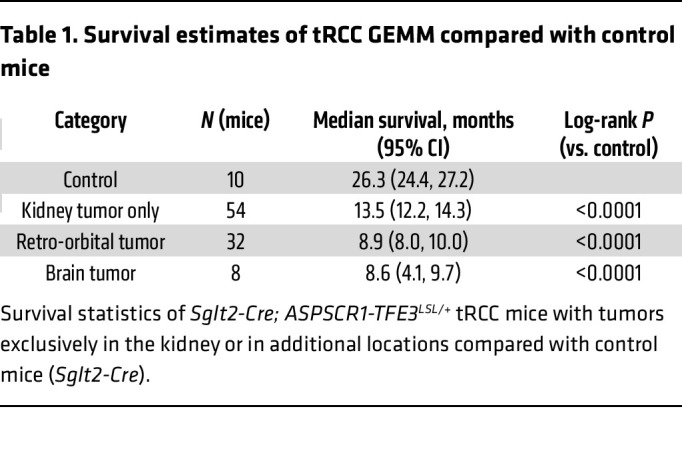
Survival estimates of tRCC GEMM compared with control mice

**Table 5:**
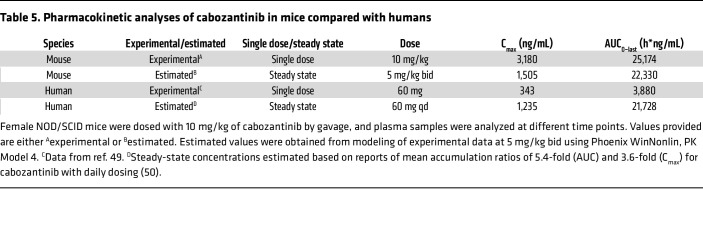
Pharmacokinetic analyses of cabozantinib in mice compared with humans

## Version 1 02/22/2024

In-Press Preview

## Version 2 04/01/2024

Electronic publication
