# Supplementary material for: Comparative genomics incorporating translocation renal cell carcinoma mouse model reveals molecular mechanisms of tumorigenesis
Source: J Clin Invest. 2024 Feb 22;134(7):e170559. doi: 10.1172/JCI170559 (PMC10977987; doi:10.1172/JCI170559)
Supplement: Supplemental data [file jci-134-170559-s166.pdf]

Comparative Genomics Incorporating Translocation Renal Cell Carcinoma Mouse Model Reveals Molecular Mechanisms of Tumorigenesis

Gopinath Prakasam[1,2], Akhilesh Mishra[1,2], Alana Christie[1,3], Jeffrey Miyata[1,2], Deyssy Carrillo[1,2], Vanina T. Tcheuyap[1,2], Hui Ye[1,2], Quyen N. Do[4], Yunguan Wang[5], Oscar Reig Torras[1,6], Ramesh Butti[1,2], Hua Zhong[1,7], Jeffrey Gagan[7], Kevin B Jones[8], Thomas J. Carroll[9], Zora Modrusan[10], Steffen Durinck[11], Mai-Carmen Requena-Komuro[1,2], Noelle Williams[12], Ivan Pedrosa[1,4,13,14], Tao Wang[1,5], Dinesh Rakheja[7], Payal Kapur[1,7,14*], James Brugarolas[1,2*]

[1]Kidney Cancer Program, Simmons Comprehensive Cancer Center, The University of Texas Southwestern Medical Center, Dallas, TX 75390, USA.

[2]Hematology-Oncology Division, Department of Internal Medicine, The University of Texas Southwestern Medical Center, Dallas, TX 75390, USA.

[3]Peter O' Donnell Jr. School of Public Health, The University of Texas Southwestern Medical Center, Dallas, TX 75390, USA.

[4]Department of Radiology, The University of Texas Southwestern Medical Center, Dallas, TX 75390, USA.

[5]Quantitative Biomedical Research Center, Department of Population and Data Sciences, The University of Texas Southwestern Medical Center, Dallas, TX 75390, USA.

[6]Department of Medical Oncology and Translational Genomics and Targeted Therapies in Solid Tumors, Institut d'Investigacions Biomèdiques August Pi I Sunyer (IDIBAPS), Hospital Clinic de Barcelona, Barcelona 08036, Spain.

[7]Department of Pathology, The University of Texas Southwestern Medical Center, Dallas, TX 75390, USA.

[8]Department of Orthopaedics and Oncological Sciences, Huntsman Cancer Institute, University of Utah, Salt Lake City, UT 84112, USA.

[9]Department of Molecular Biology and Department of Internal Medicine, University of Texas Southwestern Medical Center, Dallas, Texas, USA.

[10]Department of Microchemistry, Proteomics, Lipidomics and NGS, Genentech Inc., South San Francisco, CA 94080, USA.

[11]Department of Oncology Bioinformatics, Genentech, Inc., South San Francisco, CA 94080, USA.

[12]Department of Biochemistry, The University of Texas Southwestern Medical Center, Dallas, TX 75390, USA.

[13]Advanced Imaging Research Center, The University of Texas Southwestern Medical Center, Dallas, TX 75390, USA.

[14]Department of Urology, The University of Texas Southwestern Medical Center, Dallas, TX 75390, USA.

***Correspondence:**

Payal Kapur

Kidney Cancer Program

Department of Pathology

Division of Anatomic Pathology, UH 04.257

The University of Texas Southwestern Medical Center

6201 Harry Hines Boulevard, Dallas, TX 75390-9234

Phone: +1 (214) 633-6363

Payal.Kapur@UTSouthwestern.edu

and

James Brugarolas

Kidney Cancer Program

Department of Internal Medicine

Division of Hematology-Oncology, ND3.120C

The University of Texas Southwestern Medical Center

5323 Harry Hines Boulevard, Dallas, TX 75390-8852

Phone: +1 (214) 648-4059

James.Brugarolas@UTSouthwestern.edu

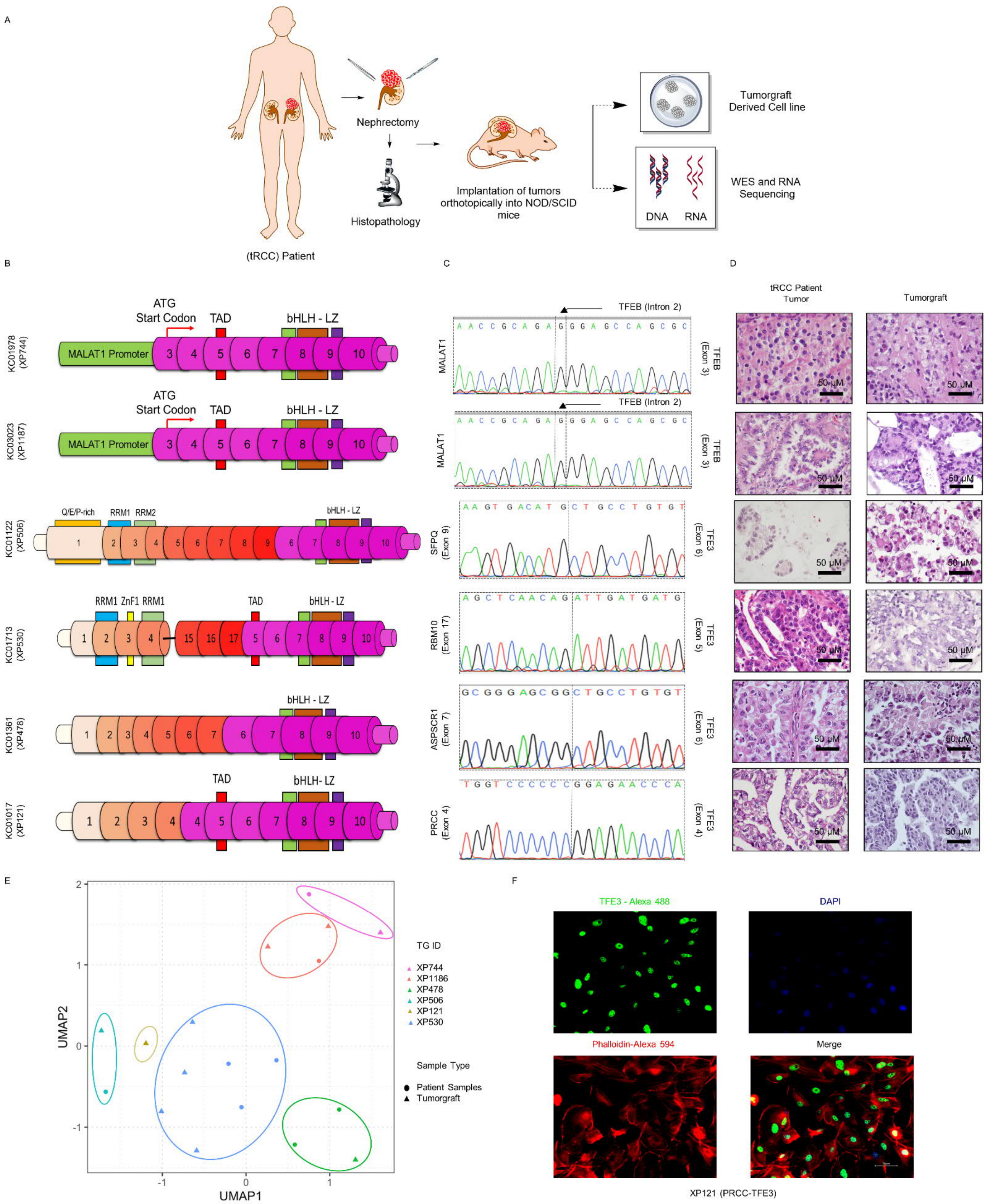

A
Nephrectomy
Histopathology
(tRCC) Patient
Implantation of tumors orthotopically into NOD/SCID mice
Tumorgraft Derived Cell line
DNA
RNA
WES and RNA Sequencing
B
KC01978 (XP744)
KC03023 (XP1187)
KC01122 (XP506)
KC01713 (XP530)
KC01361 (XP478)
KC01017 (XP121)
ATG Start Codon
TAD
bHLH - LZ
MALAT1 Promoter
Q/E/P-rich
RRM1
RRM2
ZnF1
C
TFEB (Intron 2)
MALAT1
TFEB (Exon 3)
SFPQ (Exon 9)
TFE3 (Exon 6)
RBM10 (Exon 17)
TFE3 (Exon 5)
ASPSCR1 (Exon 7)
PRCC (Exon 4)
TFE3 (Exon 4)
D
tRCC Patient Tumor
Tumorgraft
50 μM
E
UMAP1
UMAP2
TG ID
XP744
XP1186
XP478
XP506
XP121
XP530
Sample Type
Patient Samples
Tumorgraft
F
TFE3 - Alexa 488
DAPI
Phalloidin-Alexa 594
Merge
XP121 (PRCC-TFE3)

Supplemental Figure. S1. tRCC Tumorgraft Cohort. A, Schema illustrating generation and characterization of TG lines. **B,** Illustration depicting the predicted MiT/TFE gene fusion based on RNAseq. **C,** Sanger sequencing electropherograms of TG gene fusions (available in only one direction for XP121, XP478, XP744 and XP1187). **D,** Representative H&E images from tRCC in patients and their corresponding TG. **E,** UMAP representation of normalized gene expression read counts for tRCC in patients (n=8, from 5 patients) and matched TGs (n=10, from 6 cases). **F,** Immunofluorescence staining of XP121 cell line derived from TG with *PRCC-TFE3* gene fusion showing nuclear TFE3 (green) with counterstaining for phalloidin (red).

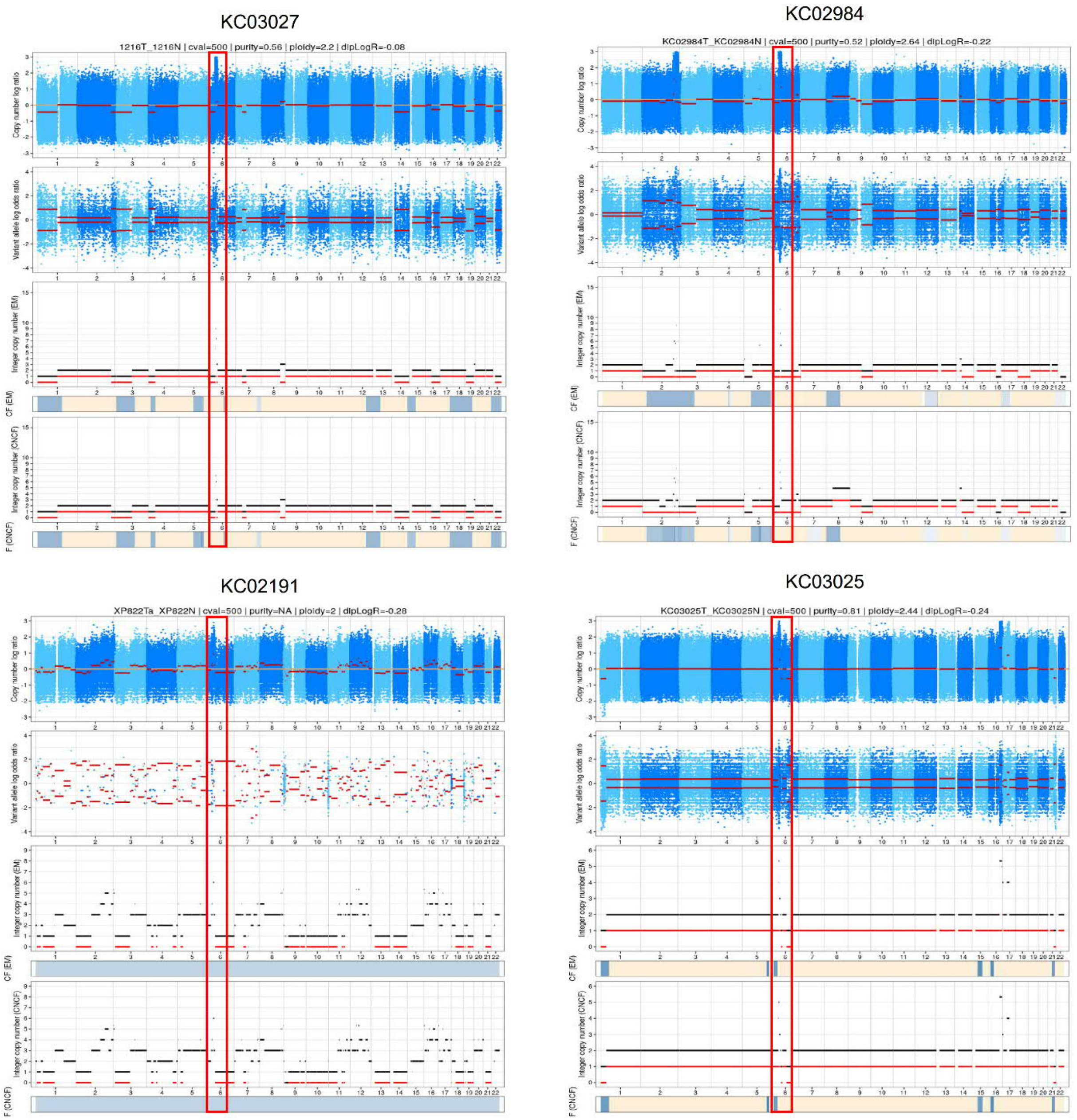

Supplemental Figure. S2. *TFEB* Amplification. Copy number analysis highlighting *TFEB* amplification in four tRCC cases.

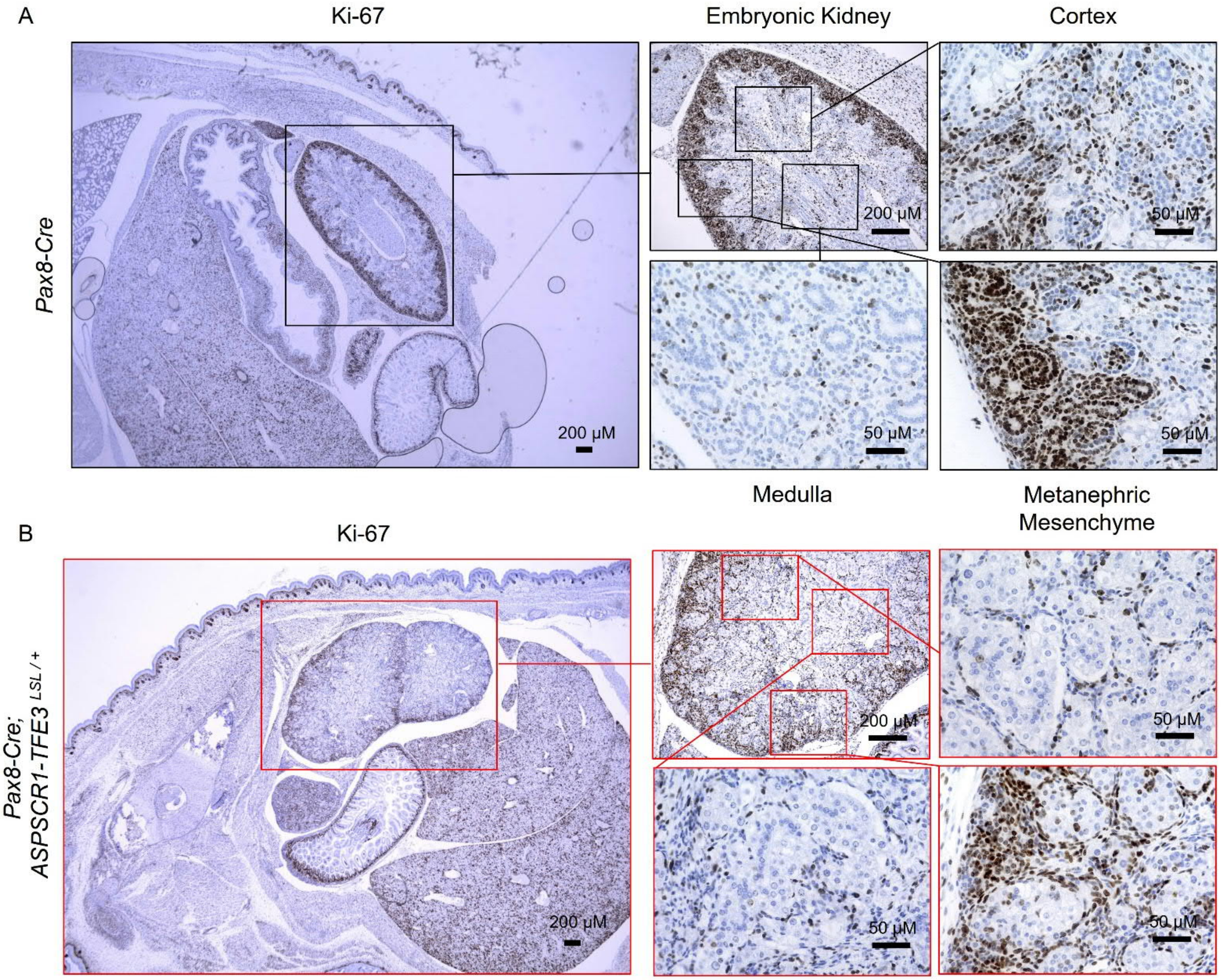

Supplemental Figure. S3. Evaluation of Cell Proliferation in *Pax8-Cre; ASPSCR1-TFE3* Dysmorphic Kidneys. A, Ki-67 staining of embryonic kidney in *Pax8-Cre* control. **B,** Ki-67 staining of embryonic kidney in *Pax8-Cre; ASPSCR1-TFE3* $^{LSL/+}$ mice. Includes images previously shown in Figure 2E.

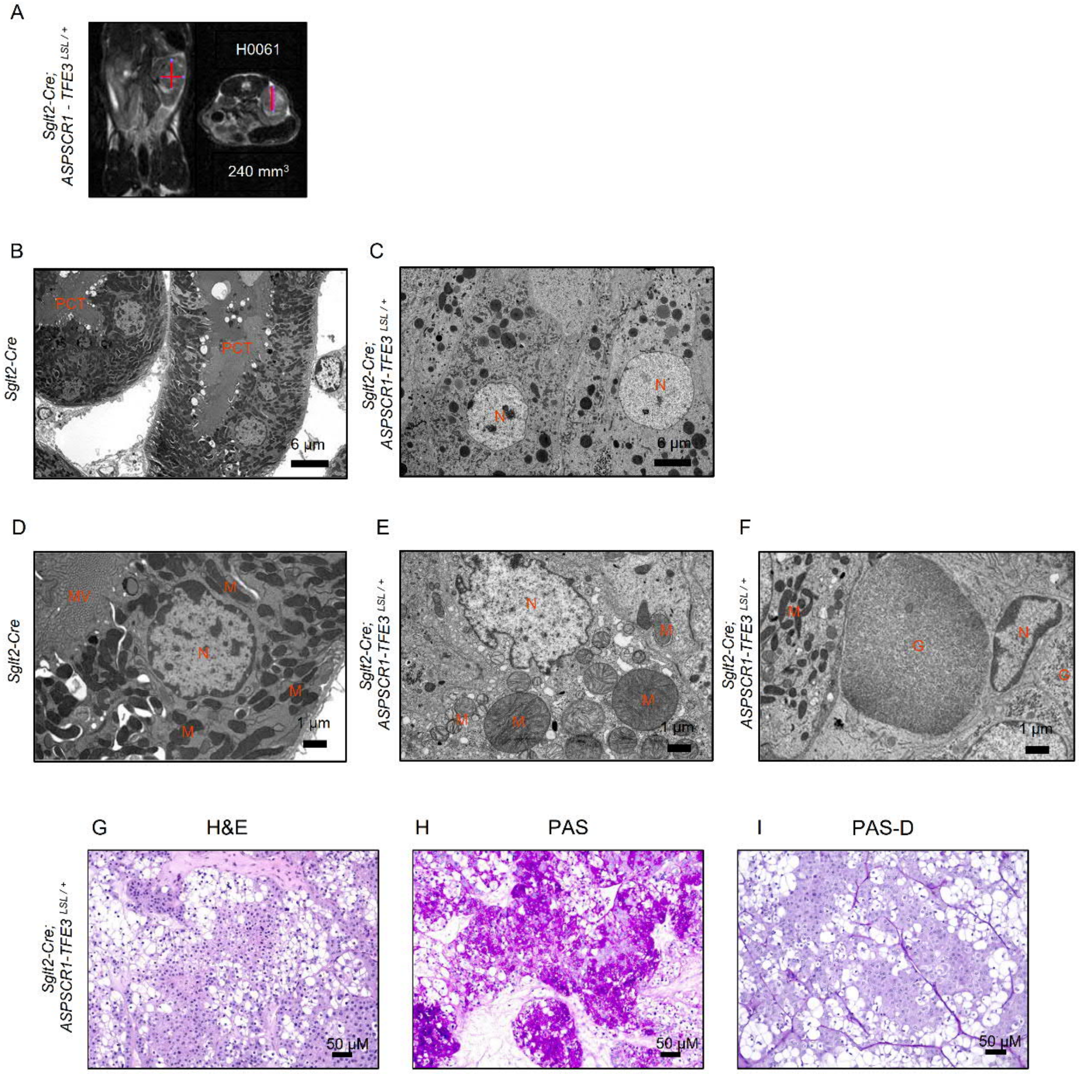

Supplemental Figure. S4. Characterization of *Sglt2-Cre; ASPSCR1-TFE3* tRCC. A, Representative MRI images of 4-month-old *Sglt2-Cre; ASPSCR1-TFE3* $^{LSL/+}$ mouse highlighting large renal mass. **B-F**, Transmission Electron Microscopy (TEM) images of kidney specimens at low **(B, C)** and high magnification **(D-F)** showing a cross-section of a renal tubule from *Sglt2-Cre* mouse kidney **(B, D)** and enlarged tumor cells in *Sglt2-Cre; ASPSCR1-TFE3* $^{LSL/+}$ tRCC with voluminous cytoplasm **(C)**, variably sized mitochondria **(E)** as well as intracellular glycogen **(F)**. **G-I,** H&E **(G)** and periodic acid-schiff (PAS) staining **(H)** with diastase to hydrolyze polysaccharides **(I)** of a representative murine tRCC tumor showing glycogen accumulation (based on n=3). G, Glycogen; M, Mitochondria; MV, Microvilli; N, Nucleus; PCT, Proximal convoluted tubule (lumen).

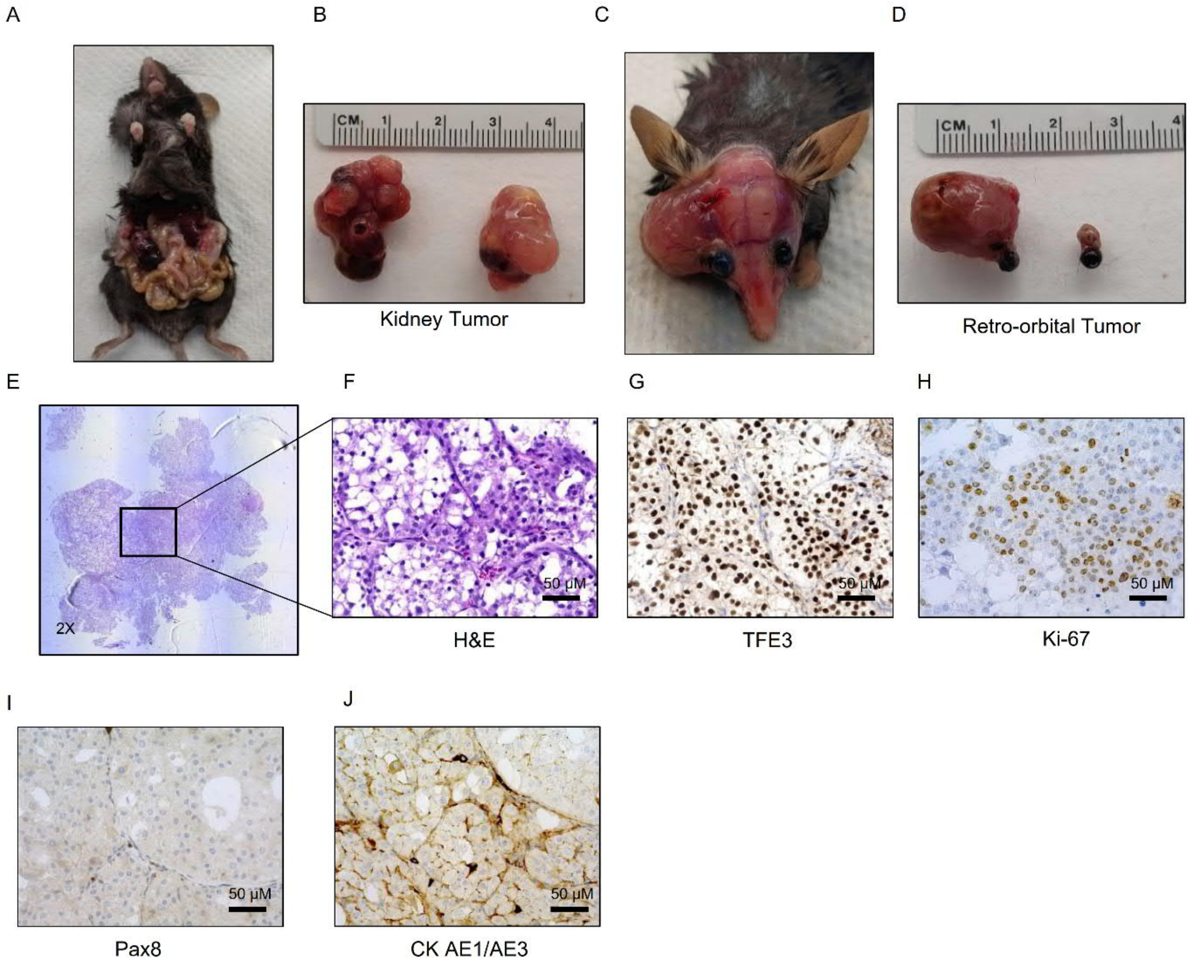

Supplemental Figure. S5. ***Sglt2-Cre; ASPSCR1-TFE3*** ***LSL/+*** **Mice Develop Retro-orbital Alveolar Soft Part Sarcoma (ASPS). A-D,** Representative gross anatomical images of *Sglt2-Cre; ASPSCR1-TFE3* *LSL/+* mouse with retro-orbital and kidney tumors. **E-F,** H&E image of representative retro-orbital tumor at lower and higher magnifications depicting pseudopapillary architecture and tumor cells with voluminous clear cytoplasm typical of ASPS. **G-J,** Immunohistochemistry for human TFE3, Ki-67, Pax8 and pan-cytokeratin (CK AE1/AE3).

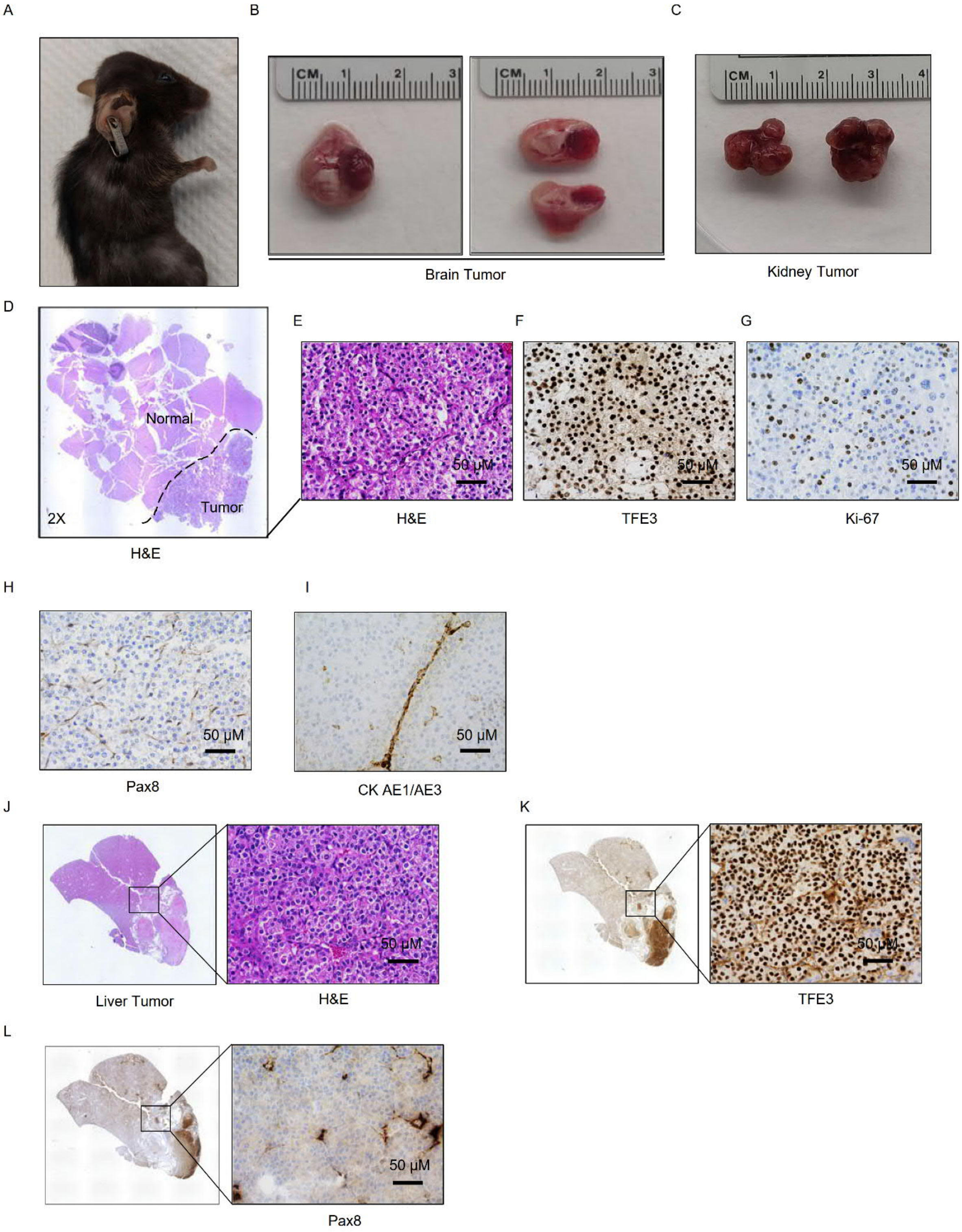
A
B
C
CM 1 2 3
Brain Tumor
CM 1 2 3 4
Kidney Tumor
D
Normal
Tumor
2X
H&E
E
50 μM
H&E
F
50 μM
TFE3
G
50 μM
Ki-67
H
50 μM
Pax8
I
50 μM
CK AE1/AE3
J
Liver Tumor
50 μM
H&E
K
50 μM
TFE3
L
50 μM
Pax8

Supplemental Figure. S6. Intracranial ASPS and Liver PEComa in *Sglt2-Cre; ASPSCR1-TFE3* $^{LSL/+}$ **Mice. A-C,** Representative images of *Sglt2-Cre; ASPSCR1-TFE3* $^{LSL/+}$ mouse with hydrocephalus due to intracranial tumor and corresponding gross anatomical images of intracranial tumor as well as multiple bilateral kidney tumors. **D-E,** H&E staining of the intracranial tumor at lower and higher magnifications. **F-I,** Immunohistochemistry for human TFE3, Ki-67, Pax8 and pan-cytokeratin. **J-L,** H&E staining of liver PEComa **(J)** with IHC for human TFE3 **(K)** and Pax8 **(L)**.

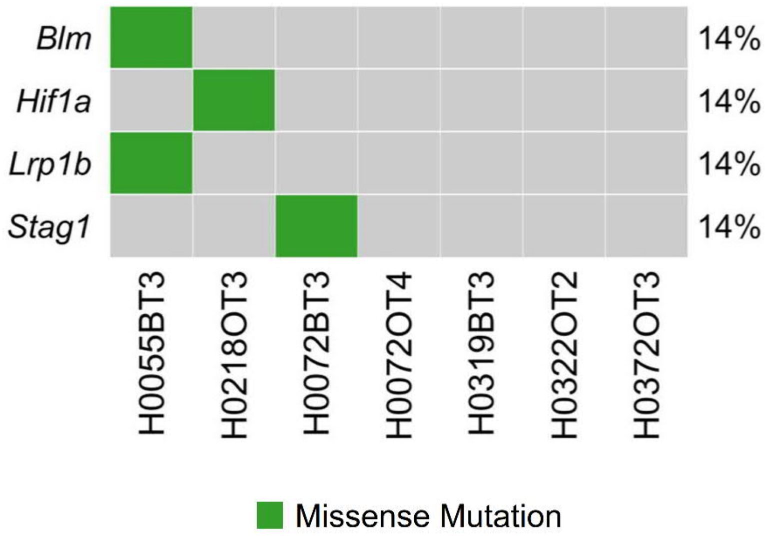

Supplemental Figure. S7. Mutational Landscape of *Sglt2-Cre; ASPSCR1-TFE3* $^{LSL/+}$ Retro-orbital and Brain Tumors. Oncoprint of *Sglt2-Cre; ASPSCR1-TFE3* $^{LSL/+}$ retro-orbital and brain tumors with somatically mutated genes listed in COSMIC.

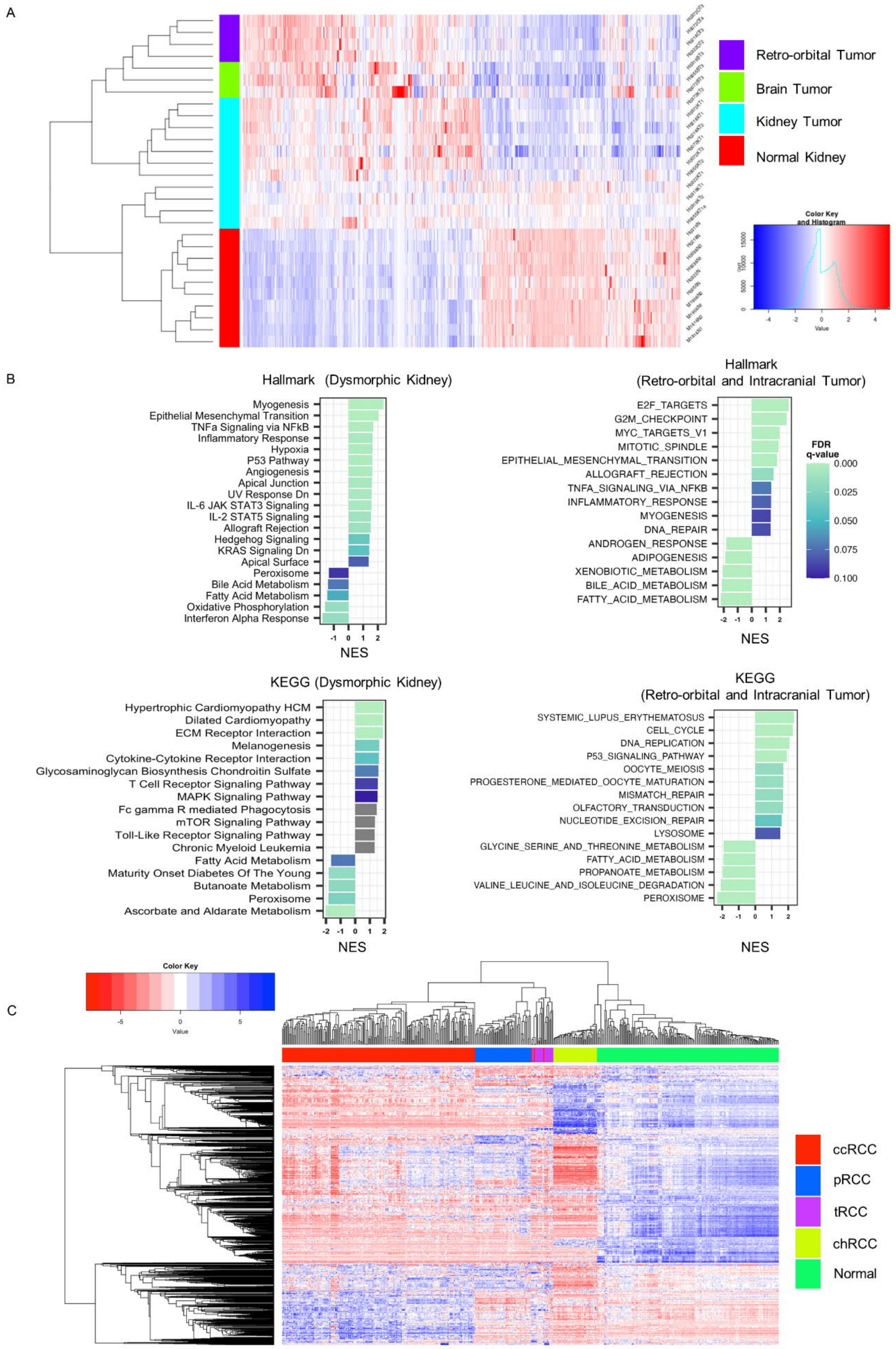

A
Retro-orbital Tumor
Brain Tumor
Kidney Tumor
Normal Kidney
Color Key and Histogram
B
Hallmark (Dysmorphic Kidney)
Myogenesis
Epithelial Mesenchymal Transition
TNFa Signaling via NFkB
Inflammatory Response
Hypoxia
P53 Pathway
Angiogenesis
Apical Junction
UV Response Dn
IL-6 JAK STAT3 Signaling
IL-2 STAT5 Signaling
Allograft Rejection
Hedgehog Signaling
KRAS Signaling Dn
Apical Surface
Peroxisome
Bile Acid Metabolism
Fatty Acid Metabolism
Oxidative Phosphorylation
Interferon Alpha Response
NES
Hallmark (Retro-orbital and Intracranial Tumor)
E2F_TARGETS
G2M_CHECKPOINT
MYC_TARGETS_V1
MITOTIC_SPINDLE
EPITHELIAL_MESENCHYMAL_TRANSITION
ALLOGRAFT_REJECTION
TNFA_SIGNALING_VIA_NFKB
INFLAMMATORY_RESPONSE
MYOGENESIS
DNA_REPAIR
ANDROGEN_RESPONSE
ADIPOGENESIS
XENOBIOTIC_METABOLISM
BILE_ACID_METABOLISM
FATTY_ACID_METABOLISM
NES
FDR q-value
KEGG (Dysmorphic Kidney)
Hypertrophic Cardiomyopathy HCM
Dilated Cardiomyopathy
ECM Receptor Interaction
Melanogenesis
Cytokine-Cytokine Receptor Interaction
Glycosaminoglycan Biosynthesis Chondroitin Sulfate
T Cell Receptor Signaling Pathway
MAPK Signaling Pathway
Fc gamma R mediated Phagocytosis
mTOR Signaling Pathway
Toll-Like Receptor Signaling Pathway
Chronic Myeloid Leukemia
Fatty Acid Metabolism
Maturity Onset Diabetes Of The Young
Butanoate Metabolism
Peroxisome
Ascorbate and Aldarate Metabolism
NES
KEGG (Retro-orbital and Intracranial Tumor)
SYSTEMIC_LUPUS_ERYTHEMATOSUS
CELL_CYCLE
DNA_REPLICATION
P53_SIGNALING_PATHWAY
OOCYTE_MEIOSIS
PROGESTERONE_MEDIATED_OOCYTE_MATURATION
MISMATCH_REPAIR
OLFACTORY_TRANSDUCTION
NUCLEOTIDE_EXCISION_REPAIR
LYSOSOME
GLYCINE_SERINE_AND_THREONINE_METABOLISM
FATTY_ACID_METABOLISM
PROPANOATE_METABOLISM
VALINE_LEUCINE_AND_ISOLEUCINE_DEGRADATION
PEROXISOME
NES
C
Color Key
Value
ccRCC
pRCC
tRCC
chRCC
Normal

Supplemental Figure. S8. Murine and Human Tumor Transcriptomic Analyses. A, Heatmap of unsupervised hierarchical clustering of differentially expressed genes from *Sglt2-Cre; ASPSCR1-TFE3* $^{LSL/+}$ tumors and normal kidney controls (*Sglt2-Cre*). **B,** Gene set enrichment analysis (Hallmark and KEGG) showing upregulated and downregulated pathways in dysmorphic kidneys from *Pax8-Cre; ASPSCR1-TFE3* $^{LSL/+}$ litters as well as in retro-orbital/ intracranial brain tumors (*Sglt2-Cre; ASPSCR1-TFE3* $^{LSL/+}$) compared to embryonic and adult kidneys, respectively. **C,** Heatmap of unsupervised hierarchical clustering of differentially expressed genes from UTSW pan-RCC cohort of ccRCC (n = 193), pRCC (n = 55), chRCC (n = 43), tRCC (n = 19) as well as normal kidney samples (n = 179). NES, normalized enrichment score; FDR, false discovery rate.

A

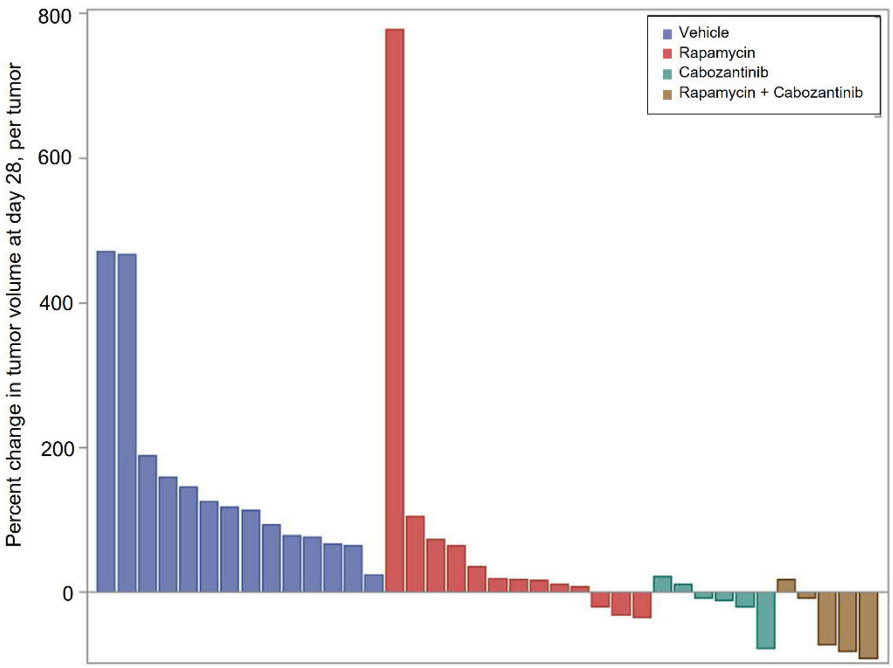

B

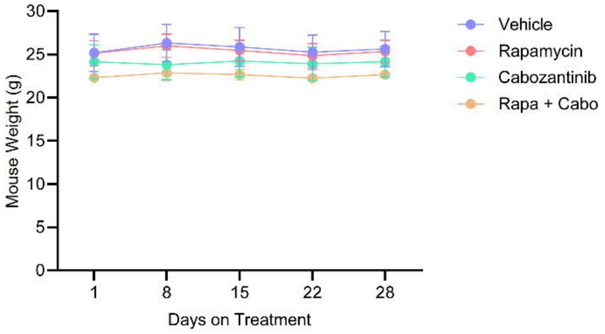

Supplemental Figure. S9. tRCC Tumor Growth Inhibition by Cabozantinib and Rapamycin. A, Waterfall plot illustrating percent change for each tumor in tRCC mice. One tumor from the vehicle group and another from the cabozantinib + rapamycin group are not included as they were only detected on the day 28 scan (25mm^3 and 16mm^3, respectively). **B,** Mouse weight measurements throughout drug trial. Error bars represent standard error of the mean (S.E.M).

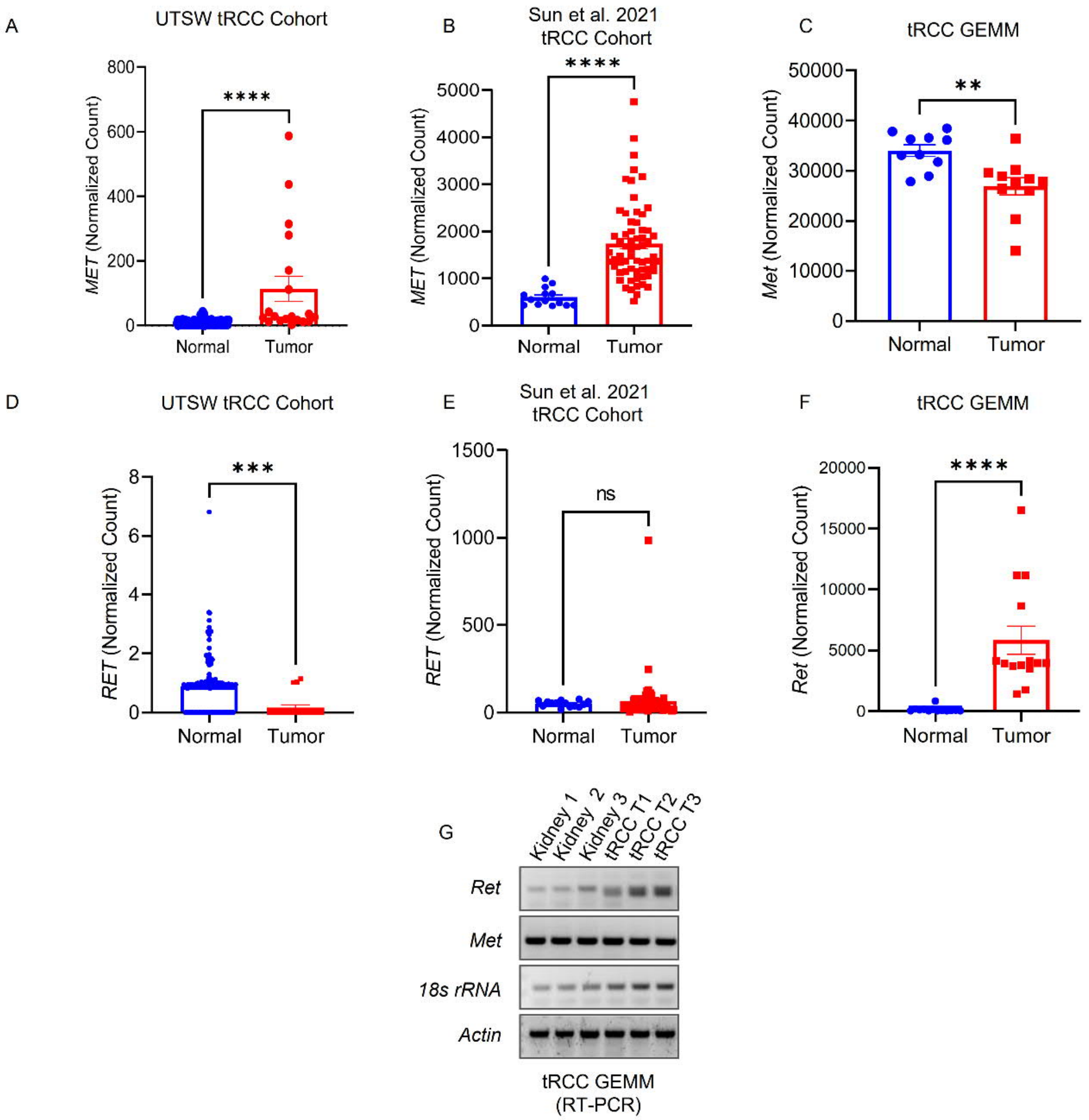

Supplemental Figure. S10. Evaluation of MET and RET in tRCC. A-F, Normalized RNA-Seq counts for *MET* **(A-C)** and *RET* **(D-F)** in human and murine tRCC cohorts as indicated. Statistical significance for differences in gene expression between normal and tumor was assessed using an unpaired Student's t-test. **G,** Real-time PCR for the indicated genes in normal kidneys from *Sglt2-Cre* mice as well as kidney tumors from *Sglt2-Cre; ASPSCR1-TFE3* $^{LSL/+}$ mice. Error bars represent mean ± standard error of the mean (S.E.M) where n ≥ 10. **, p < 0.01; ***, p <0.001; ****, p < 0.0001; ns, not significant.

Supplemental Methods

Histology and Immunohistochemistry.

Pax8-Cre; ASPSCR1-TFE3 $^{LSL/+}$ fetuses were collected from the uterus of pregnant females, fixed in 10% (v/v) buffered formalin, sectioned and embedded in paraffin following serial dehydration in ethanol. Tumors (as well as normal kidney samples) collected from the *Sglt2-Cre; ASPSCR1-TFE3* $^{LSL/+}$ mice (or controls) were fixed in buffered formalin, embedded in paraffin, and processed for downstream analysis. 4 µm thin tissue sections were stained with hematoxylin and eosin (H&E) and examined by a board-certified pathologist. Immunostaining was carried out as previously described (1, 2) using Autostainer Link 48 (Dako, Agilent Technologies). In brief, sections were air-dried over-night, deparaffinized, rehydrated and antigens were retrieved using Envision FLEX Target Retrieval Solution, High and Low pH (Dako). Primary antibodies were used as follows. Cell Signaling Technology (CST): pS6 ribosomal protein (Ser240/244) Catalog No. 5364, 1:300; Ki-67 (D3B5) Catalog No. 12202, 1:100; Cleaved Caspase 3 (Asp175) (D3E9) Catalog No. 9579, 1:600. Proteintech: PAX8 Catalog No. 10336-1-AP, 1:1000. Sigma: TFE3 (Cell Marque, Clone MRQ-37) Catalog No. 354R-14, 1:50. Thermo Fisher Scientific: CK18 (E431-1) Catalog No. MA5-14479, 1:20. Biocare: pan-cytokeratin (AE1/AE3) Catalog No. CM011A, 1:100. An Envision FLEX System (Dako, Agilent Technologies) was used following the manufacturer's instructions to detect antibody staining (Envision FLEX^{+} Rabbit linker [SM805] and Mouse linker [SM804]) except for Ki-67, where an avidin/biotin detection system (Vector Laboratories: anti-Rabbit IgG Catalog No. BA-1000) was used instead. 3,3′-diaminobenzidine chromogen was used to develop the staining and specimens were counterstained with hematoxylin. For each immunostaining run, appropriate positive and negative controls were used.

Periodic Acid-Schiff Staining.

Periodic Acid-Schiff (PAS) staining with and without diastase was carried out on paraffin-embedded sections per the manufacturer instructions (Poly Scientific R&D Corp.). Following the PAS staining, specimens were counterstained with hematoxylin.

Transmission Electron Microscopy (TEM).

tRCC mouse (*Sglt2-Cre; ASPSCR1-TFE3* $^{LSL/+}$) kidney tumor samples (n=3) and normal kidneys (n=3) from control mice (*Sglt2-Cre*) were cut into 1 mm^3 pieces and fixed with 2.5% (v/v) glutaraldehyde dissolved in 0.1M sodium cacodylate buffer. Samples were briefly rinsed in 0.1M sodium cacodylate buffer and post fixed in 0.8% potassium ferricyanide and 1% osmium tetroxide mixed in 0.1M sodium cacodylate buffer for 90 minutes at room temperature. Excess fixative was removed by rinsing in water and samples were stained with 4% uranyl acetate in 50% ethanol for 2 hours. They were dehydrated with increasing concentrations of ethanol, transitioned into a resin with propylene oxide, infiltrated with EMbed-812 resin and polymerized in a 60°C oven overnight. Blocks were sectioned with a diamond knife (Diatome) on a Leica Ultracut 7 ultramicrotome (Leica Microsystem), collected onto copper grids and stained with 2% aqueous uranyl acetate and lead citrate. Images were acquired with HT7700 transmission electron microscope (Hitachi, Chiyoda City, Tokyo, Japan) equipped with BioSptint16 CCD camera (Advanced Microscopy Techniques, Woburn, MA, USA).

Western Blot Analyses.

Tumor and normal tissues as well as tissue culture samples were homogenized in modified RIPA lysis buffer (50 mM Tris-HCl pH 7.2, 150 mM NaCl, 0.1% SDS, 0.5% sodium deoxycholate and 1% Triton X-100) for 30 minutes at 4°C supplemented with 1 mM PMSF, a protease inhibitor

mixture (Sigma-Aldrich: #P8340) and phosphatase inhibitor cocktail-2 (Sigma-Aldrich: # P5726) and cocktail-3 (Sigma-Aldrich: #P0044). The protein lysate was cleared by centrifugation at 15000 RPM for 20 minutes, and protein concentration was determined using a BCA kit (Thermo Scientific). Protein lysates were resolved by SDS-PAGE and transferred to a polyvinylidene difluoride (PVDF) membrane, which was blocked with 5% non-fat milk in phosphate-buffered saline (PBS) and probed with primary antibodies at 4°C overnight. The antibodies used were as follows: Cathepsin K (3F9) (Abcam, Catalog No. ab37259), ASPSCR1 (Bethyl, Catalog No. A302-351A), RPS6 (Bethyl, Catalog No. A300-557A), 4E-BP1 (53H11) (Cell Signaling Technology (CST), Catalog No. 9644), Phospho-4E-BP1 (T37/46) (236B4) (CST, Catalog No. 2855), Atg3 (CST, Catalog No. 3415), Atg5 (D5F5U) (CST, Catalog No. 12994), Atg7 (D12B11) (CST, Catalog No. 8558), Atg12 (D88H11) (CST, Catalog No. 4180), Beclin-1 (D40C5) (CST, Catalog No. 3495), LAMTOR1 (D11H6) (CST, Catalog No. 8975), LC3A/B (D3U4C) (CST, Catalog No.12741), Phospho-p70 S6 Kinase1/2 (T421/S424) (CST, Catalog No. 9204), Phospho-S6 Ribosomal Protein (S240/244) (CST, Catalog No. 2215), RagA/B (D8B5) (CST, Catalog No. 4357), RagC (D8H5) (CST, Catalog No. 9480) and SQSTM1/p62 (CST, Catalog No. 5114), α-Tubulin (Sigma-Aldrich, Catalog No. T5168), TFE3 (MRQ-37) (Sigma-Aldrich, Catalog No. 354R-14). The following secondary antibodies were used, HRP Goat-anti-mouse IgG (Thermo Fisher Scientific, Catalog No. 31430) and HRP Goat-anti-rabbit- IgG (Thermo Fisher Scientific, Catalog No. 31460). Protein bands were visualized using a chemiluminescence kit (Bio-Rad) and ChemiDoc Imaging Systems (Bio-Rad).

Mammalian Cell Culture and Immunofluorescence Microscopy.

XP121 cells derived from a tRCC TG were cultured in Dulbecco’s Modified Eagle Medium with F12 Glutamax (DMEM/F12 from Life Technologies, Catalog No. 11320033) supplemented with

10% fetal bovine serum (FBS from Life Technologies, Catalog No. A5256701), 1X penicillin–streptomycin (Sigma Aldrich, Catalog No. P4458), 1% MEM nonessential amino acids (Gibco, Catalog No. 11140050), 5 ng/mL EGF (Thermo Fisher Scientific, Catalog No.PHG0313) and 0.4 µg/mL hydrocortisone (Sigma Aldrich, Catalog No. H0396) in a humidified incubator (Nuaire) at 37 °C with 5% CO_2. XP121 cells were grown in coverslips, fixed in 10% formalin buffered in PBS for 20 min and then blocked/ permeabilized (PBS containing 5 % BSA and 0.1% Triton X-100) for 1 hour at room temperature. A primary antibody (anti-Rabbit TFE3; Sigma, Catalog No. 354R-14, 1:200 dilution) was added overnight at 4°C. After washing, the coverslips were incubated with anti-rabbit Alexa Fluor 488 (Thermo Fisher Scientific, Catalog No. A-11008) and Alexa Fluor™ 594 Phalloidin (Thermo Fisher Scientific, Catalog No. P36931) at 37°C. Coverslips were then washed and mounted on a glass slide with ProLong® Gold Antifade Reagent with DAPI (Thermo Fisher Scientific Catalog No. P36931). Images were captured using a Keyence epi-fluorescence microscope (Catalog No. BZ-X700).

Nucleic Acid Extraction for Next Generation Sequencing.

Samples were submitted to the New York Genome Center (NYGC) for nucleic acid extraction as well as Whole Exome Sequencing (WES) and RNA-sequencing (RNA-Seq). 6 *Sglt2-Cre; ASPSCR1-TFE3* $^{LSL/+}$ mice were used for WES/RNA-Seq studies encompassing 24 samples: 11 kidney tumors (one kidney tumor sample [H0218KT1] was dropped from downstream analyses, because of poor DNA quality); 4 retro-orbital tumors; 3 brain tumors as well as 6 non-transformed kidney samples. In addition, RNA isolation and RNA-Seq were performed on 6 normal kidney samples from 3 *Sglt2-Cre* control mice. Kidney samples from 5 *Pax8-Cre; ASPSCR1-TFE3* $^{LSL/+}$ fetus and 3 *Pax8-Cre* or *ASPSCR1-TFE3* $^{LSL/+}$ age-matched controls were submitted for RNA-Seq.

All samples were from formalin-fixed and paraffin-embedded (FFPE) tissues (**Supplemental Table S5**).

For the clinical cohort, untreated FFPE samples from 13 patients and both fresh frozen (FF) as well as FFPE samples for 4 patients were submitted for WES and RNA-Seq. WES and RNA-Seq of frozen tissue was available on 13 cases from previous studies (3, 4) (**Supplemental Table S2**). Similarly, RNA-Seq and WES data for the tRCC tumorgrafts (n = 6) were also from a previous study (5). DNA and RNA were co-extracted from frozen human tRCC samples using an all-Prep DNA/RNA Mini kit (Qiagen, #80204) according to the manufacturer's recommendations. DNA QC included quantification by fluorescence using the Quant-iT PicoGreen dsDNA assay (Life Technologies, #P7589) on a Spectramax fluorometer (Molecular Devices) and DNA integrity using a High Sensitivity Genomic DNA Fragment Analysis kit (Agilent, #DNF-488-1000) in a Fragment Analyzer (Agilent). RNA QC was determined using a fluorescence-based Quant-it Ribogreen Assay kit (Life Technologies, #R11490) and integrity was confirmed with a fragment analyzer using the HS-RNA FA Analysis kit (Agilent, #DNF-472-0500).

DNA and RNA were extracted from mouse and human FFPE samples in parallel using an in-house method developed by the NYGC. This method uses a thermostable protease in a novel digestion buffer with saponified medium-chain triglycerides for the efficient separation of high-quality DNA and RNA. Solid phase reversible immobilization beads (Beckman Coulter, #B23319) were used to purify nucleic acid. To repair some of the DNA damage resulting from fixation and storage, the DNA was subjected to a prolonged enzymatic process using a PreCR Repair Mix (NEB, #M0309L).

Whole Exome Sequencing.

Whole exome sequencing was carried out at the New York Genome Center (NYGC) employing the following methods for DNA extracted from human FF and FFPE tissues as well as mouse FFPE samples, with minor modifications according to tissue type. The following methods were used for DNA extracted from human FF and FFPE tissues as well as mouse FFPE samples with minor modifications according to tissue type. A Hamilton Microlab STAR Liquid Handling System was used to normalize extracted DNA. For FFPE samples, 100-200ng of normalized DNA was fragmented using a low-volume AFA plate; for FF samples, 200ng-1500ng of normalized DNA was fragmented using an AFA plate. A Covaris LE220 was used according to the manufacturer's recommendations. Library preparation was performed on the SciClone NGS workstation (PerkinElmer). To create libraries suitable for Agilent targeted enrichment capture, fragmented samples were processed with a KAPA HyperPrep kit (Roche, #KK8504). SureSelectXT adapters (Agilent, #G9611C) were ligated to the DNA fragments using KAPA reagents. The pre-capture libraries were quantified with a Quant-iT PicoGreen dsDNA assay and the fragment size distribution was assessed on a Fragment Analyzer. Exome capture for human and mouse samples was performed with a pre-capture library using the SureSelectXT Human All Exon V6+COSMIC probe set (Agilent, #5190-9308) as well as SureSelectXT Mouse All Exon probe set (Agilent, #G7550) following manufacturer recommendations. Enriched fragments were uniquely indexed during the final amplification process. Final libraries were quantified by the Quant-iT PicoGreen dsDNA assay and the fragment size distribution was assessed on a Fragment Analyzer. Sequencing was performed on a NovaSeq 6000 instrument (Illumina) using the v1 S2 200-cycle reagent kit (Illumina, #20012861). On average, ~60 million paired end (2 × 125 bp) reads per sample were generated.

RNA Sequencing.

The following protocol was used by the New York Genome Center (NYGC) for whole transcriptome sequencing for both human FF and FFPE as well as for mouse FFPE samples with minor variations according to tissue type. Normalization for the total isolated RNA was carried out in a Hamilton Microlab STAR Liquid Handling System. Library preparation was performed on a SciClone NGS workstation (PerkinElmer). RNA samples were processed using a Kapa Stranded Total RNA-seq Library Preparation Kit (Roche, #KK8484) without depletion of ribosomal RNA. The library preparation followed the manufacturer's recommended protocol for processing degraded RNA with minor modifications to create libraries suitable for Agilent targeted enrichment capture. Following the first and second strand generation, SureSelectXT adapters (Agilent, #G9611C) were ligated using KAPA reagents. The pre-capture libraries were quantified with a Quant-iT PicoGreen dsDNA assay and the fragment size distribution was assessed on a Fragment Analyzer. Exome capture was performed with a minimum of 500ng of the pre-capture library using the SureSelectXT Mouse All Exon probe set (Agilent, #G7550) or SureSelectXT Human All Exon V6+COSMIC probe set (Agilent, Cat. #5190-9308) following manufacturer's recommendations. Enriched fragments were uniquely indexed during the final amplification process. The final libraries were quantified by a Quant-iT PicoGreen dsDNA assay and the fragment size distribution was assessed on a Fragment Analyzer. Sequencing was performed on a NovaSeq 6000 instrument (Illumina) using the v1 S2 200-cycle reagent kit (Illumina, #20012861). From mouse tissue, on average ~65 million paired-end reads (2 × 100 bp) per sample were generated. In humans, ~35 million paired-end (2 × 50 bp) reads per sample were generated on average. For both WES and RNA-Seq files, the FastQ ID was generated as per the following format

"Customer provided 'Client ID'_Illumina barcode sequence_flow cell ID_flow cell lane number_demux attempt".

Human and Mouse WES Analysis.

One sample from human (KC01122) and mouse (H0218KT1) tRCC tumors was dropped from WES analyses due to their poor read quality. Mutation calling of human samples was carried out using a previously published pipeline (6). Stringency was assessed by comparing the results with analyses independently carried out by NYGC (New York Genome Center) using their in-house pipeline (data not shown). Mouse tRCC WES genomic analysis was carried out by NYGC. Raw WES data for tumor and matched normal tissues were quality-checked using FastQC (7) and MultiQC (8). Using a Burrows-Wheeler Aligner (BWA), exome-seq reads were aligned to the respective reference genomes, human GRCh37 (hg19) and mouse GRCm38 (mm10) (9). To mark duplicate reads, NovoSort was used. Base quality score recalibration and local realignment around indels were achieved via a Genome Analysis Toolkit (GATK) (10). Additional quality controls were carried out using Picard. Sample mix-ups and cross-individual contamination were evaluated using Conpair. Strelka (11), muTect (12) and LoFreq (13) were employed to identify somatic Single Nucleotide Variants (SNVs), while for the detection of indels, Strelka, along with the somatic versions of Pindel (14) and Scalpel (15), were utilized. snpEff (16) in mouse and SnpEff, snpSift, and GATK Variant Annotator module in humans were used to annotate SNPs, indels as well as protein sequence changes. Silent, intronic, untranslated, and intergenic mutations were filtered out.

We focused our analysis on cancer-related genes based on the Catalogue of Somatic Mutations in Cancer (COSMIC) (17). We specifically considered genes with a minimum of 5 altered reads within tumors and a minimum variant allele frequency (VAF) of 5% (as shown in Supplemental

Table S4). To create the OncoPrint for human and mouse mutation data, we used the R packages ComplexHeatmap (18) and Maftools (19), respectively. Stringent criteria were applied to reduce the rate of false positives in oncoprints requiring a minimum of 15 altered reads within tumor samples and a minimum VAF of 15%. Additionally, the human oncoprint features only genes mutated in at least two tumors, whereas for the smaller mouse dataset, single instances of gene mutation were included. Potential discrepancies among samples from the same source were manually resolved using the Integrative Genomics Viewer (IGV) (20). We used Facets R package (v.0.6.2) (21) to reconstruct allele-specific copy-number profiles from WES bam files of tumor and paired normal samples.

RNA-Sequencing Data Analysis.

Two non-tumor kidney samples from *Sglt2-Cre; ASPSCR1-TFE3* $^{LSL/+}$ mice (H0072N and H0372N) were dropped due to poor read quality. FastQC (7) and MultiQC (8) were used to run quality control tests on raw RNA-Seq data from mice and humans. rRNA abundance was measured by Bowtie2 (22). RNA-seq reads were aligned to the appropriate reference genome: Human (GRCh37 (hg19) and Mouse GRCm38 (mm10) using STAR (v2.5.2a) (23) with the following parameters "--runMode alignReads --runThreadN 1 --genomeDir refGenome --genomeLoad LoadAndRemove --readFilesIn /fastq/Sample.R1.fastq.gz /fastq/Sample.R2.fastq.gz --readFilesCommand zcat --outReadsUnmapped None --outSAMstrandField intronMotif --outSAMattributes All --outSAMunmapped Within --alignIntronMax 200000 --alignMatesGapMax 200000 --chimSegmentMin 15 --chimJunctionOverhangMin 15." FeatureCounts (v1.4.3-p1) with parameters "-s 2 -a gencode.annotation.gtf –o output.txt file.bam" were then used to measure expression levels. The human and mouse genome annotation file employed by featureCounts was downloaded from GENCODE. Downstream analyses were

performed using the R computing environment (version 4.1.0). DESeq2 (24) was used to normalize featureCounts and to assess fold change as well as statistical significance of differentially expressed genes. Genes with an absolute log2 fold change ≥ 1 or < -1 and with a p adj < 0.05 were considered statistically significant. The DESeq2 normalized expression data were also used to perform Gene Set Enrichment Analysis (GSEA) on Hallmark and KEGG pathways. All the PCA plots were generated using the R/Bioconductor package pcaExplorer (25) considering all the genes in the dataset. Heatmaps (unsupervised clustering) were generated using heatmap.2 function from gplots R-package considering top 5000 differentially expressed genes. Volcano plots were generated using VolcaNoseR web tool (26).

Gene Fusion Prediction.

Gene fusions were predicted using STAR-Fusion (v1.9.0) (27) with the parameters "--genome_lib_dir genome_lib --left_fq "Align.R1.fastq.gz" --right_fq "Align.R2.fastq.gz" --output_dir star_fusion_denovo --denovo_reconstruct --FusionInspector validate --examine_coding_effect." STARFusion identified MiT/TFE gene fusions in 20 out of 30 cases in our institutional tRCC cohort. However, in two cases (KC03033 and KC03003) only the reciprocal translocation product (TFE3-ASPSCR1) was detected. That these cases harbor an ASPSCR1-TFE3 gene fusion was supported by FISH/Cytogenetics and TFE3 IHC. In cases where STARFusion failed, we performed analyses with manual inspection of discordant reads flanking key exons of MiT/TFE family members using the IGV viewer leading to the identification of gene fusions in 4 more cases (KC03001, KC03004, KC03034 and KC00987). We utilized the RCircos tool, an R package, to generate a Circos plot (28).

RT-PCR and MiT/TFE Gene Fusion Validation.

Frozen tissues were homogenized, and total RNA was isolated using Invitrogen PureLink RNA mini kit. For FFPE clinical samples, RNA was isolated using RNeasy FFPE Kit (Qiagen) according to the manufacturer instructions. 1 - 2 μg of total RNA was reverse transcribed using a High-Capacity cDNA Reverse Transcription kit (Applied Biosystems™). With the exception of XP121, where the RNA was extracted from a cell line derived from TG, for all other cases, RNA was obtained directly from tumorgrafts to confirm the presence of gene fusions. To validate each gene-fusion, primers were designed to amplify sequences flanking the predicted chromosomal breakpoint. PCR amplified bands were resolved by agarose gel electrophoresis. Purified and eluted products were then submitted for Sanger sequencing. Sequencing was conducted at the UT Southwestern Medical Center McDermott Center Sequencing Core facility using Life Technologies® (LT) Dye Terminator 3.1 chemistry and 3730XL Genetic Analyzers. All fusions were validated by bidirectional Sanger sequencing except for the TG lines XP121, XP478, XP744 and XP1187 which were only available for one direction. Primers are available upon request.

MRI Imaging.

MRI images were captured as previously described (2). In brief, 1T Desktop MR scanner (M2 Compact, Aspect Imaging) with a mouse volume coil was used (prone position). General T1-weighed and T2-weighted images were acquired with a spin echo sequence (SE; TR/TE = 350/13 ms) and a fast spin echo (FSE; TR/TE = 2500/80 ms) sequence, respectively. Diameter-based tumor size measurements were employed to assess tumor size and volume. The craniocaudal diameter and lateral diameters were determined on coronal T2-weighted images while the anteroposterior diameter was determined in axial T2-weighted images. Diameter-based tumor volume was then computed by multiplying the three dimensions. In tumors exhibiting a large cystic

component (i.e., following the signal intensity of simple fluid), a secondary diameter-based tumor volume was calculated after excluding the cyst.

Cabozantinib Pharmacokinetics Study.

6-8 week old NOD-SCID mice were used for pharmacokinetic (PK) analysis. Mice were administered a single dose of 10 mg/kg of cabozantinib by gavage (0.2 ml/mouse formulated in 0.5% methylcellulose/0.1% Tween 80 in dH20). Animals were euthanized at different timepoints with CO_2 and blood was immediately collected by cardiac puncture. Plasma was recovered by centrifugation of ACD (acidified citrate dextrose) treated blood for 10' at 9,600 x g in a standard centrifuge. Plasma aliquots were cleared of protein by mixing with a 2-fold volume of methanol containing 0.15% formic acid as well as 112.5 ng/ml n-benzylbenzamide internal standard followed by vortexing and centrifugation (to pellet precipitated protein). Cabozantinib levels in the supernatant were measured by LC-MS/MS using a Sciex 4000 QTRAP™ coupled to a Shimadzu Prominence HPLC. Standards, prepared by spiking purchased blank plasma (BioIVT, Westbury, NY) with varying concentrations of cabozantinib were processed following the same procedure. Chromatography conditions were as follows. Buffer A consisted of water + 0.1% formic acid and Buffer B consisted of methanol + 0.1% formic acid. The column flow rate was 1.5 ml/min using an Agilent C18 XDB, 5 micron packing 50 X 4.6 mm size column. The gradient conditions were 0.01 – 1.0 min 3% B, 1.0 - 1.5 min gradient to 100% B, 1.5 - 3.5 min 100% B, 3.5 - 3.6 min gradient to 3% B, 3.6 - 4.0 3% B. Cabozantinib was detected in MRM mode by following the precursor to fragment ion transition 502.2 to 307.1. N-benzylbenzamide (transition 212.1 to 91.1) was used as internal standard. A value 3-fold above the signal obtained from blank plasma was designated as the limit of detection (LOD). The limit of quantitation (LOQ) was defined as the lowest concentration at which back calculation yielded a concentration within 20% of theoretical

and which was above the LOD. The LOQ for cabozantinib was 0.5 ng/ml. In general, back calculation of points yielded values within 15% of theoretical over five orders of magnitude (0.5 ng/ml to 5000 ng/ml). PK parameters were calculated in sparse sampling mode using the noncompartmental analysis tool of Phoenix WinNonlin™ (Certara Corporation, Mountain View, CA) and then modeled using compartmental analysis (PK Model 4).

Drug Trials.

6-12-month-old *Sglt2-Cre; ASPSCR1-TFE3* $^{LSL/+}$ mice were evaluated for kidney tumors by MRI at baseline (0 day) and allocated into 4 treatment groups (Vehicle, Rapamycin, Cabozantinib and Rapamycin + Cabozantinib). Rapamycin was dissolved in 5% ethanol, 5% PEG400, 5% Tween 80, and 85% D5W and administered intraperitoneally (I.P) at 0.5 mg/kg every 48 h, a regimen mimicking human exposure (1). The same carrier was used for the vehicle group (5% ethanol, 5% PEG400, 5% Tween 80, and 85% D5W) administered intraperitoneally every 48 h. Cabozantinib was dissolved in MCT solution (0.5% methylcellulose, 0.5% Tween 80) and administered by oral gavage at 5 mg/kg every 12 h, a regimen mimicking human exposure. Tumor growth was monitored by MRI at the end of trial. Mouse weight was recorded weekly to assess drug toxicity. Mice were euthanized and tumor tissue samples were collected as previously described (2).

References

1. Sivanand S, Pena-Llopis S, Zhao H, Kucejova B, Spence P, Pavia-Jimenez A, et al. A validated tumorgraft model reveals activity of dovitinib against renal cell carcinoma. *Sci Transl Med.* 2012;4(137):137ra75.
2. Gu YF, Cohn S, Christie A, McKenzie T, Wolff N, Do QN, et al. Modeling Renal Cell Carcinoma in Mice: Bap1 and Pbrm1 Inactivation Drive Tumor Grade. *Cancer Discov.* 2017;7(8):900-17.
3. Durinck S, Stawiski EW, Pavia-Jimenez A, Modrusan Z, Kapur P, Jaiswal BS, et al. Spectrum of diverse genomic alterations define non-clear cell renal carcinoma subtypes. *Nat Genet.* 2015;47(1):13-21.
4. Kim K, Zhou Q, Christie A, Stevens C, Ma Y, Onabolu O, et al. Determinants of renal cell carcinoma invasion and metastatic competence. *Nat Commun.* 2021;12(1):5760.
5. Elias R, Tcheuyap VT, Kaushik AK, Singla N, Gao M, Reig Torras O, et al. A renal cell carcinoma tumorgraft platform to advance precision medicine. *Cell Rep.* 2021;37(8):110055.
6. Lu T, Wang S, Xu L, Zhou Q, Singla N, Gao J, et al. Tumor neoantigenicity assessment with CSiN score incorporates clonality and immunogenicity to predict immunotherapy outcomes. *Sci Immunol.* 2020;5(44).
7. Andrews S. Babraham Bioinformatics, Babraham Institute, Cambridge, United Kingdom; 2010.
8. Ewels P, Magnusson M, Lundin S, and Kaller M. MultiQC: summarize analysis results for multiple tools and samples in a single report. *Bioinformatics.* 2016;32(19):3047-8.

9. Li H, and Durbin R. Fast and accurate short read alignment with Burrows-Wheeler transform. *Bioinformatics.* 2009;25(14):1754-60.

10. McKenna A, Hanna M, Banks E, Sivachenko A, Cibulskis K, Kernytsky A, et al. The Genome Analysis Toolkit: a MapReduce framework for analyzing next-generation DNA sequencing data. *Genome Res.* 2010;20(9):1297-303.

11. Saunders CT, Wong WS, Swamy S, Becq J, Murray LJ, and Cheetham RK. Strelka: accurate somatic small-variant calling from sequenced tumor-normal sample pairs. *Bioinformatics.* 2012;28(14):1811-7.

12. Cibulskis K, Lawrence MS, Carter SL, Sivachenko A, Jaffe D, Sougnez C, et al. Sensitive detection of somatic point mutations in impure and heterogeneous cancer samples. *Nat Biotechnol.* 2013;31(3):213-9.

13. Wilm A, Aw PP, Bertrand D, Yeo GH, Ong SH, Wong CH, et al. LoFreq: a sequence-quality aware, ultra-sensitive variant caller for uncovering cell-population heterogeneity from high-throughput sequencing datasets. *Nucleic Acids Res.* 2012;40(22):11189-201.

14. Ye K, Schulz MH, Long Q, Apweiler R, and Ning Z. Pindel: a pattern growth approach to detect break points of large deletions and medium sized insertions from paired-end short reads. *Bioinformatics.* 2009;25(21):2865-71.

15. Narzisi G, O'Rawe JA, Iossifov I, Fang H, Lee YH, Wang Z, et al. Accurate de novo and transmitted indel detection in exome-capture data using microassembly. *Nat Methods.* 2014;11(10):1033-6.

16. Cingolani P, Platts A, Wang le L, Coon M, Nguyen T, Wang L, et al. A program for annotating and predicting the effects of single nucleotide polymorphisms, SnpEff: SNPs in

the genome of Drosophila melanogaster strain w1118; iso-2; iso-3. *Fly (Austin).* 2012;6(2):80-92.

17. Sondka Z, Bamford S, Cole CG, Ward SA, Dunham I, and Forbes SA. The COSMIC Cancer Gene Census: describing genetic dysfunction across all human cancers. *Nat Rev Cancer.* 2018;18(11):696-705.

18. Gu Z, Eils R, and Schlesner M. Complex heatmaps reveal patterns and correlations in multidimensional genomic data. *Bioinformatics.* 2016;32(18):2847-9.

19. Mayakonda A, Lin DC, Assenov Y, Plass C, and Koeffler HP. Maftools: efficient and comprehensive analysis of somatic variants in cancer. *Genome Res.* 2018;28(11):1747-56.

20. Robinson JT, Thorvaldsdottir H, Wenger AM, Zehir A, and Mesirov JP. Variant Review with the Integrative Genomics Viewer. *Cancer Res.* 2017;77(21):e31-e4.

21. Shen R, and Seshan VE. FACETS: allele-specific copy number and clonal heterogeneity analysis tool for high-throughput DNA sequencing. *Nucleic Acids Res.* 2016;44(16):e131.

22. Langmead B, and Salzberg SL. Fast gapped-read alignment with Bowtie 2. *Nat Methods.* 2012;9(4):357-9.

23. Dobin A, Davis CA, Schlesinger F, Drenkow J, Zaleski C, Jha S, et al. STAR: ultrafast universal RNA-seq aligner. *Bioinformatics.* 2013;29(1):15-21.

24. Love MI, Huber W, and Anders S. Moderated estimation of fold change and dispersion for RNA-seq data with DESeq2. *Genome Biol.* 2014;15(12):550.

25. Marini F, and Binder H. pcaExplorer: an R/Bioconductor package for interacting with RNA-seq principal components. *BMC Bioinformatics.* 2019;20(1):331.

26. Goedhart J, and Luijsterburg MS. VolcaNoseR is a web app for creating, exploring, labeling and sharing volcano plots. *Sci Rep.* 2020;10(1):20560.

27. Haas BJ, Dobin A, Li B, Stransky N, Pochet N, and Regev A. Accuracy assessment of fusion transcript detection via read-mapping and de novo fusion transcript assembly-based methods. *Genome Biol.* 2019;20(1):213.

28. Zhang H, Meltzer P, and Davis S. RCircos: an R package for Circos 2D track plots. *BMC Bioinformatics.* 2013;14:244.
