# Supplementary material for: Comparative genomics incorporating translocation renal cell carcinoma mouse model reveals molecular mechanisms of tumorigenesis
Source: J Clin Invest. 2024 Feb 22;134(7):e170559. doi: 10.1172/JCI170559 (PMC10977987; doi:10.1172/JCI170559)

Uncropped/unedited versions of all gel and blot images, related to Figure 3D

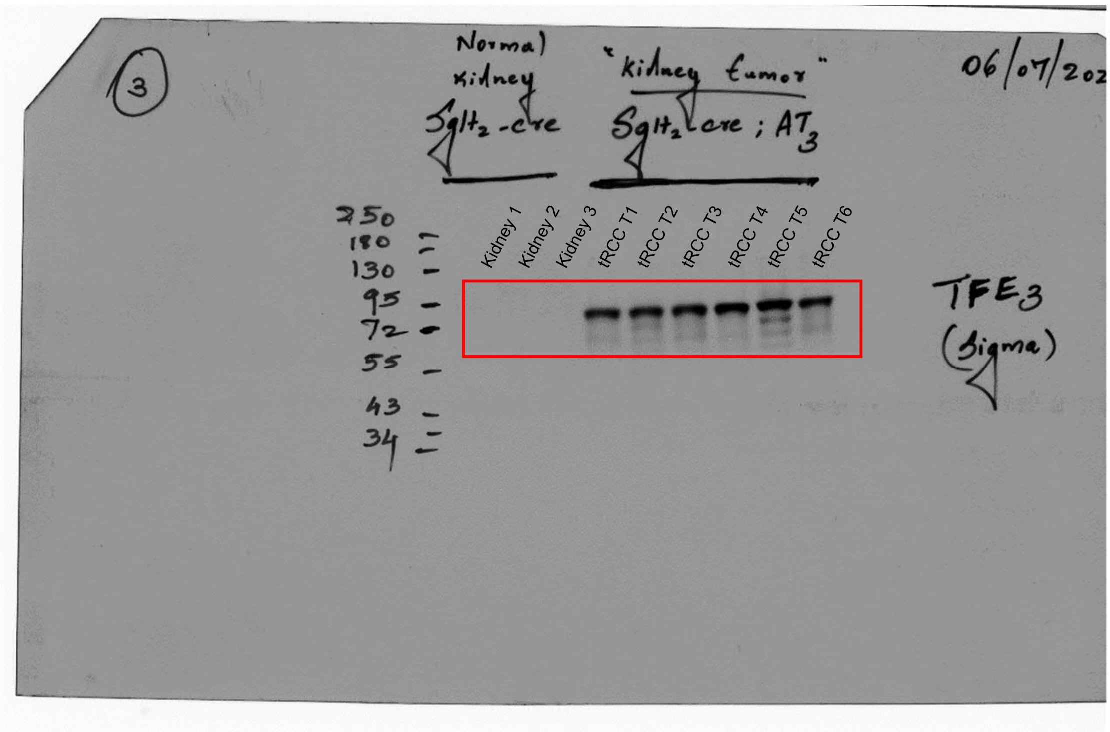

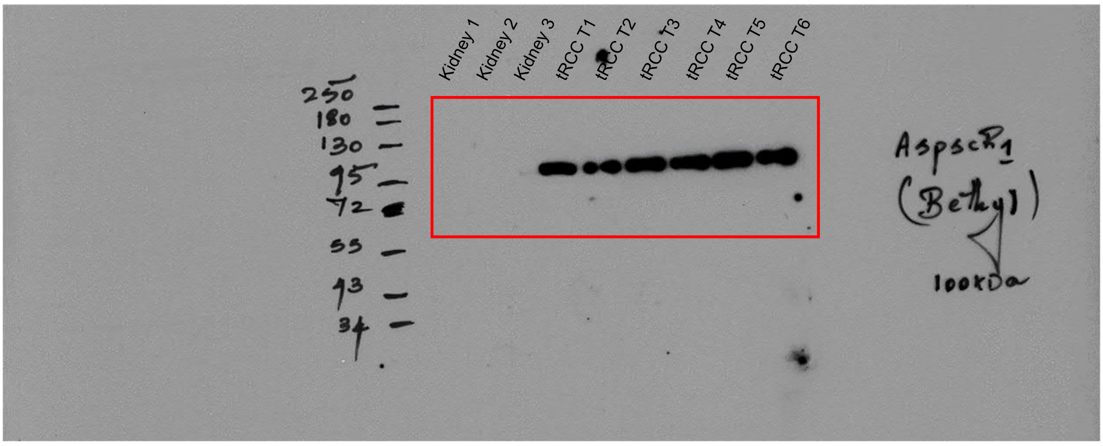

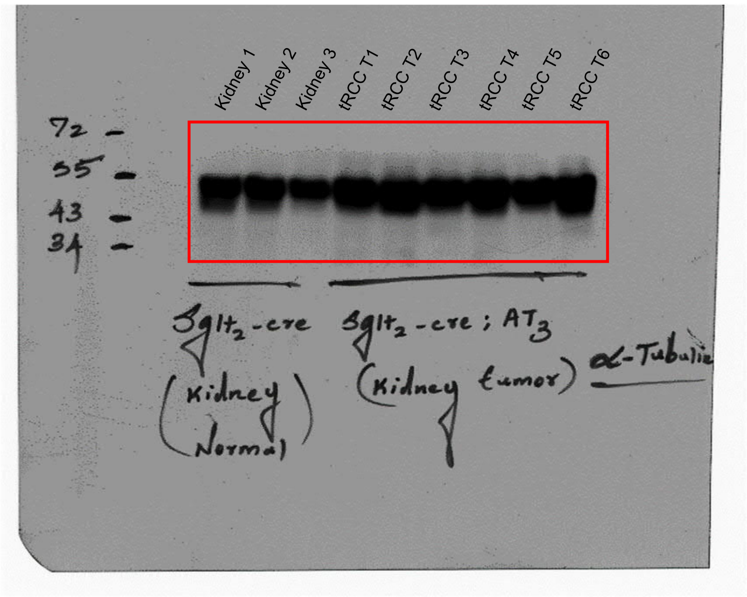

Uncropped/unedited versions of all gel and blot images, related to Figure 6C

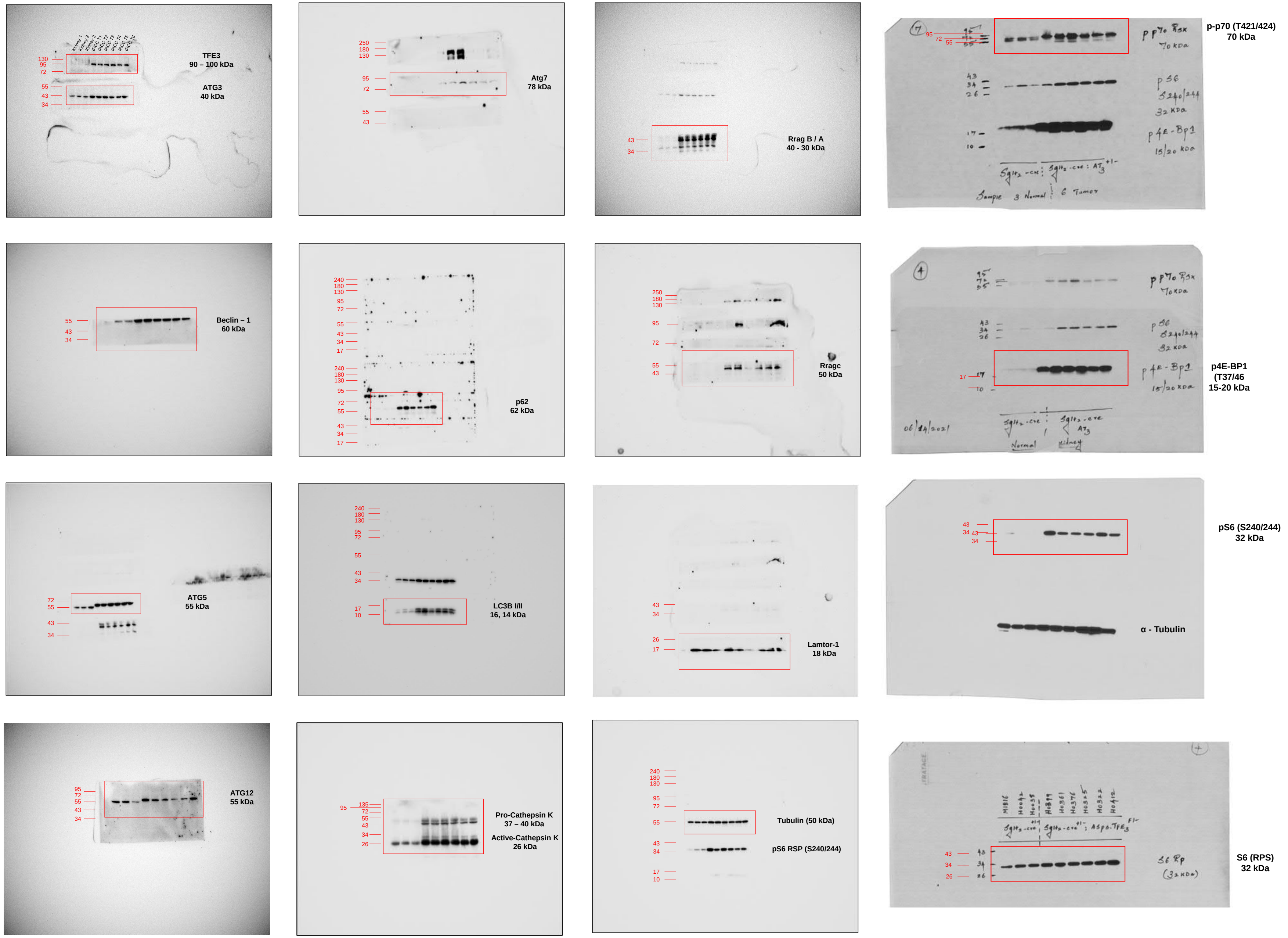

Uncropped/unedited versions of all gel and blot images, related to Figure 6J

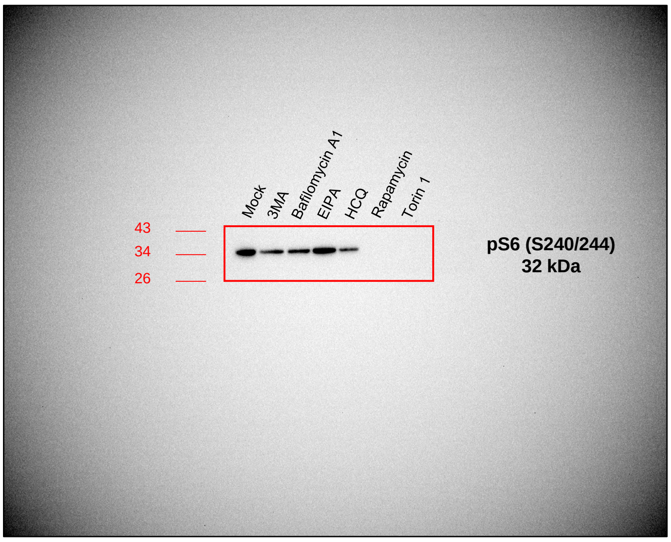

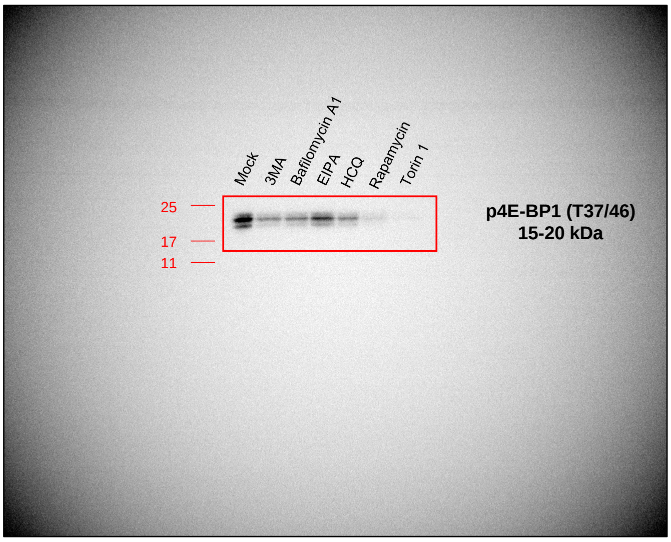

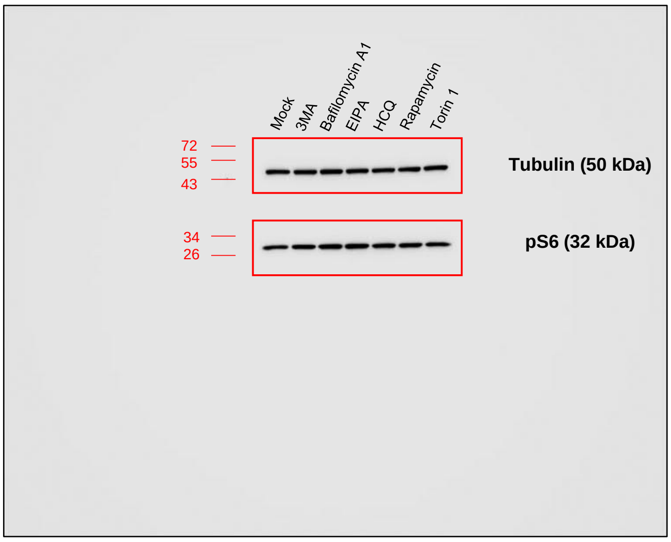

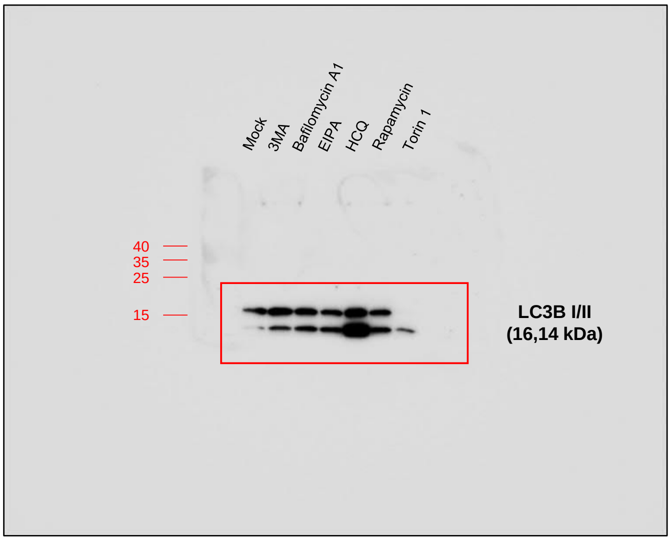

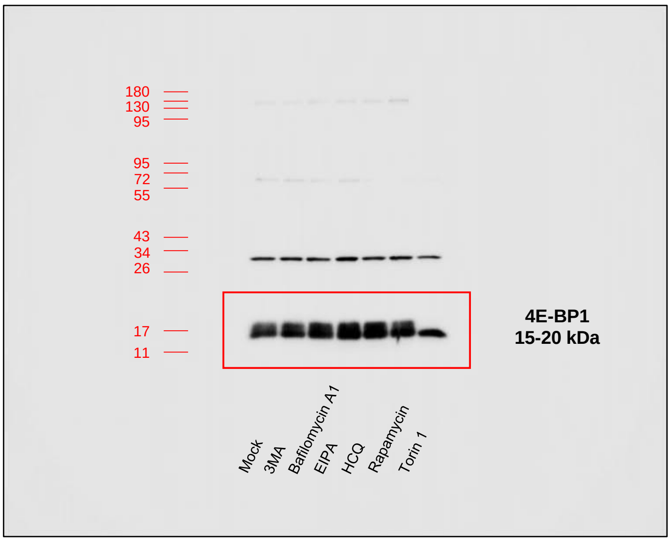

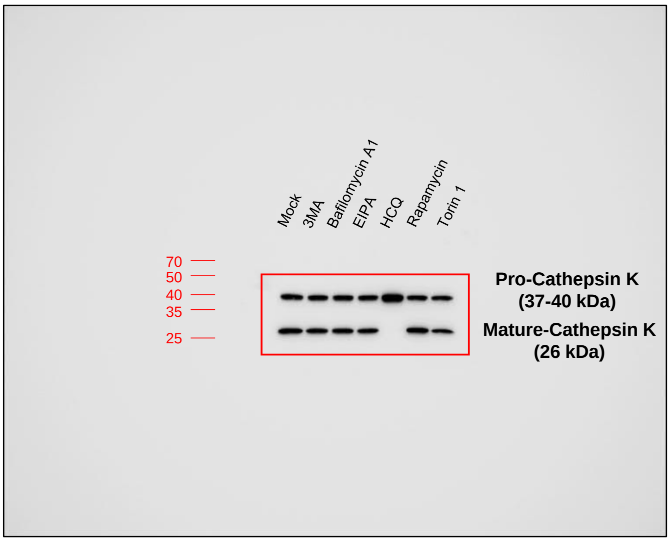

Uncropped/unedited versions of all gel and blot images, related to Supplementary Figure S10G

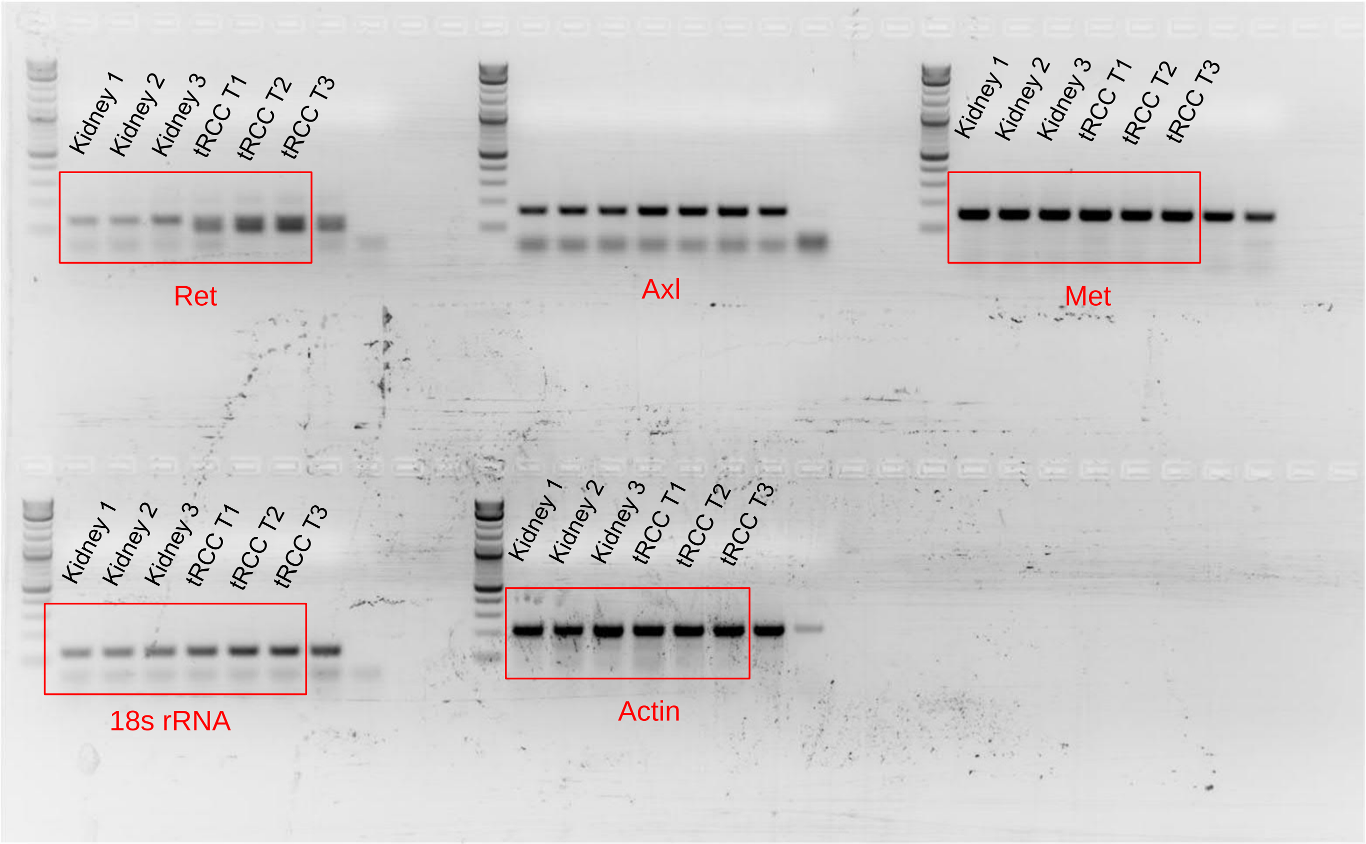

Supplement: Unedited blot and gel images [file jci-134-170559-s167.pdf]
